# Access to Self-Assembled Poly(2-Oxazoline)s through Cationic Ring Opening Polymerization-Induced Self-Assembly (CROPISA)

**DOI:** 10.1021/acs.macromol.5c01599

**Published:** 2025-07-14

**Authors:** James Lefley, Steven Huband, C. Remzi Becer

**Affiliations:** † Department of Chemistry, 2707 University of Warwick, Coventry CV4 7AL, U.K.; ‡ X-ray Diffraction RTP, Department of Physics, 2707 University of Warwick, Coventry CV4 7AL, U.K.

## Abstract

Self-assembled block copolymers provide wide range of opportunities in encapsulation and release of active compounds in aqueous and nonaqueous systems. However, developing new polymeric systems that have the precise ratio of blocks and backbone structure has been a challenge where every single methyl group has an enormous effect on the final self-assembled structure. Herein, we present a synthetic route to access sphere, worm, and vesicle shaped self-assembled nanostructures via cationic ring opening polymerization-induced self-assembly (CROPISA) method in nonaqueous media using 2-alkyl-2-oxazolines. This study successfully produced a broad range of nanostructures through careful manipulation of the solvophobicity of the core-forming block. Therefore, poly­(isostearyl-2-oxazoline) was selected as the soluble stabilizing block and CROPISA was conducted at 20 wt % solids content in *n*-heptane. Although initial attempts using PEtOx as the core-forming block yielded only spherical micelles, switching to a lower *T*_g_ and more solvophilic poly­(2-propyl-2-oxazoline) core-forming block allowed access to higher-order structures such as worms and vesicles for the first time. Finally, the thermoresponsive properties of the resulting worm organogel further demonstrated the potential for stimuli-responsive poly­(2-oxazoline) based materials in nonaqueous applications.

## Graphical abstract

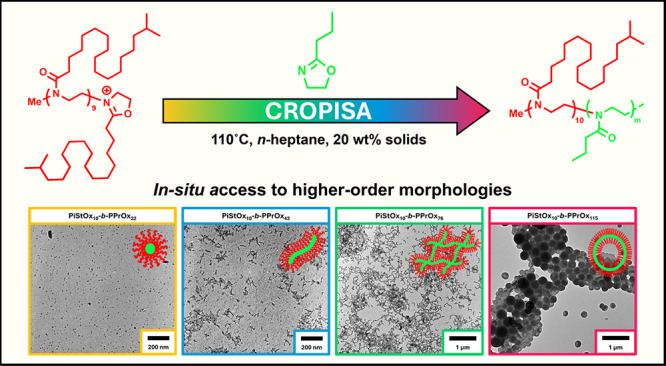

## Introduction

Self-assembly is a phenomenon widely observed in nature where molecules can spontaneously organize into functional structures and plays a crucial role in biological systems such as cell membranes and protein folding. In material science, controlled self-assembly is highly sought after to mimic nature and enable advanced applications in drug delivery, biomimetic systems, and optoelectronics.^1–3^ Understanding the driving forces behind self-assembly has allowed researchers to develop innovative nanomaterials with tailored properties for use within these fields of research. Polymerization-induced self-assembly (PISA) has emerged as a highly efficient technique for the preparation of polymeric nanostructures, offering significant advantages over traditional methods like solvent exchange and solvent-free approaches.^4^ Unlike these conventional techniques, PISA enables the in situ formation of finely tuned nanostructures by providing exceptional control over size and morphology. Additionally, PISA facilitates the preparation of nanostructures at much higher solution concentrations (up to 50 wt %) compared to the more dilute systems required by conventional methods (0.1–1.0 wt %).^5^ As a result, these advantages have prompted extensive research into PISA. Practical applications include the in situ generation of oil-soluble spheres for lubricant additives^6–9^ and the in situ encapsulation of active pharmaceutical ingredients (APIs)^10,11^ or enzymatic catalysts^12,13^ in nanocapsules, highlighting the potential of PISA to advance the synthesis of next-generation oil additives, drug delivery systems, and biomimetic artificial cells. PISA exploits the solubility differences between two polymer blocks in a medium that selectively favors one. Initially, a soluble polymer block, or ‘macroinitiator,’ is synthesized to act as the stabilizing block in the self-assembly process. By extending this stabilizing block with a solvophilic monomer, which forms a solvophobic polymer in the medium, block copolymer self-assembly occurs. By systematically varying the block length ratios and solids content in the PISA formulation, nanostructures with near-monodisperse size distributions and distinct morphologies can be precisely targeted.^4^

To date, most PISA studies have been conducted using aqueous RAFT dispersion polymerization, with significant contributions from research groups like Armes,^14–17^ Pan,^18–20^ Tan,^21–23^ Zetterlund,^24–27^ and many more. Other than RAFT, PISA has been realized using other controlled radical polymerization mechanisms such as copper-mediated reversible deactivation radical polymerization (Cu-mediated RDRP) and nitroxide-mediated polymerization (NMP). Early Cu-mediated RDRP PISA studies by Kim^28^ and Pan^29^ focused on core cross-linked micelles via ATR­(PISA) but the use of high copper catalyst concentrations made purification of the nanostructures a significant challenge thus limiting its potential industrial application, especially in biomedicine. Matyjaszewski’s group later overcame these issues by using ATRP-ICAR^30^ and ARGET-ATRP^31^ with lower catalyst levels, achieving polymeric nanostructures via PISA. Although successful PISA using SET-LRP was also reported,^32,33^ Cu-mediated PISA struggles to form higher-order morphologies beyond spheres due to the complex need for both copper and ligand species at the polymerization site, particularly within nanoparticle cores. Beyond Cu-mediated RDRP, Charleux and co-workers have published some innovative work on NMPISA via dispersion or emulsion NMP methodologies.^34–38^

However, the significant lack of NMPISA studies can be attributed to slow polymerization kinetics consequently needing higher temperatures and prolonged reaction times to achieve high monomer conversions.^39^ Ring-opening polymerization-induced self-assembly (ROPISA) offers a highly efficient alternative to radical-based mechanisms for synthesizing BCP nanostructures from cyclic peptide and cyclic ester monomers. A key advantage of ROPISA is the potential for creating biodegradable and biocompatible BCPs compared to CRP techniques that utilize acrylic and methacrylic monomers. Notable examples include the first ROPISA of α-amino acid N-carboxyanhydrides (NCA) using a PEG_45_-NH_2_ macroinitiator reported by Jiang et al.^40^ Grazon et al. then adapted this procedure giving the first aqueous ROPISA, which produced polypeptide-based nanostructures where the secondary structures of the polypeptides dictated the morphology of the nanostructures.^41,42^ Beyond *N*-carboxyanhydrides, cyclic ester monomers in ROPISA enable integration with crystallization-driven self-assembly (CDSA), producing crystalline or semicrystalline polyester blocks. This synergy forms the basis of polymerization-induced crystallization-driven self-assembly (PI-CDSA), a nascent field that merges two techniques to create rigid, anisotropic nanostructures with precise dimensional control.^43–47^ However, the adoption of PI-CDSA using ROP is limited, with few publications to date,^48–50^ due to stringent synthesis conditions like the need for ultradry reagents and specific monomer/solvent/catalyst combinations.^51^ Such conditions preclude the use of living ionic polymerization mechanisms in aqueous PISA, although nonaqueous PISA remains viable with several reports citing the use of living anionic polymerization to achieve diverse nanostructure morphologies.^43,45,52^

Poly­(2-oxazoline)­s (POx) are a class of important biocompatible and highly functionalizable polymers that are synthesized via cationic ring opening polymerization (CROP).^53^ The increasing utilization of POx in diverse applications underscores its functional versatility and adaptability to different technological needs.^54–59^ In the biomedical field, the self-assembling capabilities of POx are particularly valued for creating stable and efficient drug delivery systems. Research is ongoing to enhance the physicochemical properties of POx to improve encapsulation efficiency and release profiles, essential for therapeutic applications.^60–62^ In terms of in situ generation of POx-based nanostructures in aqueous media, few examples exist. Delaittre and co-workers reported the use of a PEtOx-based macro-RAFT agent for the chain extension of 2-Hydroxypropyl methacrylate (HPMA) in to produce in situ POx-based nanostructures in water.^63^ Alternatively, Kempe and co-workers adopted a heat-triggered CDSA strategy to produce rigid all-POx nanorods in aqueous solution.^64,65^ However, due to the inherent incompatibility of ionic polymerization and water, the direct preparation of all-POx nanostructures in aqueous solution is unattainable. However, the synthesis of all-POx nanostructures in nonaqueous media may potentially offer new application areas for POx-based materials. To our surprise, up until very recently, there were no publications detailing a PISA study whereby an all-oxazoline block copolymer (BCP) is synthesized via sequential monomer addition in a selective, nonaqueous medium for one block and inducing in situ self-assembly. Lusiani et al. recently reported the first example of cationic ring opening polymerization induced self-assembly (CROPISA) of block and gradient copolymers of PEHOx-PPhOx in *n*-dodecane forming dispersions of spheres and short worm-like nanostructures.^66^ The formation of worm-like nanostructures for a BCP with a relatively high *T*_g_ core-forming block was impressive, although a full range of nanostructure morphologies were not achieved most likely due to the rigid nature of the PPhOx core. With this in mind, we aimed to explore CROPISA further to see if higher-order morphologies, such as vesicles, could be unlocked by tuning the core-forming block using commercially available 2-oxazoline monomers.

Herein, we describe CROPISA using a long alkyl chain 2-oxazoline monomer forming the oil soluble block (PiStOx) and employing solvophobic monomers EtOx or PrOx producing amphiphilic PiStOx-*b*-PEtOx and PiStOx-*b*-PPrOx BCPs in *n*-heptane at 20 wt % solids (Scheme 1). Comprehensive kinetic studies were performed and analyses via dynamic light scattering (DLS), small-angle X-ray scattering (SAXS), and transmission electron microscopy (TEM) revealed diverse nanostructures. Notably, PiStOx-*b*-PEtOx formed kinetically trapped spheres even with highly asymmetric block ratios, while substituting PEtOx with PPrOx unlocked higher order morphologies such as worms and vesicles. Though only temporarily, as the nanostructures were shown to be colloidally unstable.

**Scheme 1:**
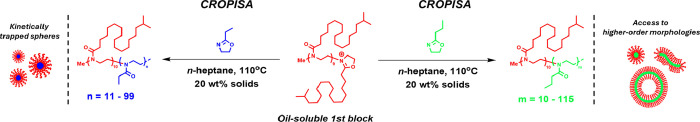
. Experimental Design for the Synthesis of PiStOx-*b*-PEtOx and PiStOx-*b*-PPrOx Block Copolymers via CROPISA in *n*-Heptane

## Experimental Section

### Materials

2-Ethyl-2-oxazoline 99+% (Acros Organics, EtOx) was dried over calcium hydride and distilled under reduced pressure prior to use. 2-isostearic-2-oxazoline (iStOx) was provided by Infineum UK and was distilled and stored under N_2_ prior to use. Methyl p-toluenesulfonate 98% (Aldrich, MeTos) was distilled under reduced pressure and stored under nitrogen. *n*-heptane (anhydrous, 99%) and *n*-dodecane (≥99%) were purchased from Sigma-Aldrich.

### Synthesis of 2-Propyl-2-oxazoline (PrOx)

Butyronitrile (40 mL, 459.56 mmol, 1 equiv), 2-amino ethanol (41.61 mL, 689.34 mmol, 1.5 equiv), and Zn­(OAc)_2_ 2 H_2_O (2.0175 g, 9.19 mmol, 0.02 equiv) as the catalyst were transferred into a round-bottom flask equipped with a magnetic stirring bar and heated to reflux at 130 °C overnight. Subsequently, the yellow-orange reaction mixture was cooled down to room temperature and 100 mL of DCM were added to the flask. The organic layer was washed with 100 mL of water (×2) and 100 mL of brine (×2) and then dried over MgSO_4_. After filtration, the solvent was evaporated under reduced pressure, and the resulting yellow oil was purified by distillation under vacuum at 50–60 °C, yielding a colorless liquid (14.32 g, 26.8% yield).

### Kinetic Investigation of the CROP of iStOx in *n*-Heptane 30 wt % Solids

To a large 20 mL degassed vial equipped with a stirrer bar, iStOx (5.00 g (5.50 mL), 16.15 mmol, 60 eqv), MeOTs (41 μL, 0.27 mmol, 1 eqv) and *n*-heptane (17 mL) were added and stirred for 10 min while being purged with N_2_. 3.0 mL of this reaction solution was taken and added to 6 separate vials microwave vials equipped with stirrer bars. Each vial was degassed for 15 min and reacted for 30, 45, 60, 120, 180, 240 min. Each polymerization was quenched with 1.0 mL of piperidine and samples were taken for ^1^H NMR and GPC analysis.

### Kinetic Investigation of the CROPISA of PiStOx-*b*-PEtOx in *n*-Heptane

First, a stock solution of iStOx (8 g (8.80 mL), 25.85 mmol, 10 Eqv), MeOTs (0.48g (0.39 mL), 2.58 mmol, 1 Eqv), and *n*-heptane (19.789 g (28.93 mL)) were added to a 50 mL Schlenk flask under N_2_. This stock solution was stored at −20 °C and used for all other CROPISA reactions. Periodic ^1^H NMR analysis of this stock solution was carried out to ensure the monomer had not reacted. In total, the stock solution was kept in the freezer and used up within a 3-week period with no signs of monomer conversion. Similarly, a second stock solution of EtOx (8.01 g (8.2 mL), 80.77 mmol) and *n*-heptane (36.44 g (53.28 mL) were added to a second Schlenk flask and kept under N_2_. 1.19 mL of the iStOx stock solution (iStOx (0.81 mmol, 10 Eqv), MeOTs (0.081 mmol, 1 Eqv), *n*-heptane (0.904 mL)) was then added to 9 separate vials and purged with N_2_ for 15 min. All 9 vials were placed in the oil bath and reacted for 1 h at 110 °C. After, the vials were removed from the oil bath and allowed to cool to room temperature before a N_2_ line was inserted to each vial. A small aliquot was taken for ^1^H NMR and GPC analysis of the PiStOx block. To each vial, 6.14 mL of the EtOx stock solution (EtOx (8.08 mmol, 100 Eqv) and *n*-heptane (5.33 mL)) was added to each vial and further reacted for 15, 30, 60, 90, 120, 180, 240, 360, 420 min. After, a small aliquot was taken for ^1^H NMR and GPC analysis of the block copolymer.

### Kinetic Investigation of the CROPISA of PiStOx-*b*-PPrOx in *n*-Heptane

The same iStOx stock solution from the previous kinetic study was used. Similarly, a second stock solution of PrOx (9.14g (9.14 mL), 80.77 mmol) and *n*-heptane (40.98g (59.91 mL) were added to a second Schlenk flask and kept under N_2_. 1.19 mL of the iStOx stock solution (iStOx (0.81 mmol, 10 Eqv), MeOTs (0.081 mmol, 1 Eqv), *n*-heptane (0.904 mL)) was then added to 9 separate vials and purged with N_2_ for 15 min. All 9 vials were placed in the oil bath and reacted for 1 h at 110 °C. After, the vials were removed from the oil bath and allowed to cool to room temperature before a N_2_ line was inserted to each vial. A small aliquot was taken for ^1^H NMR and GPC analysis of the PiStOx block. To each vial, 6.91 mL of the EtOx stock solution (EtOx (8.08 mmol, 100 Eqv) and *n*-heptane (5.99 mL)) was added to each vial and further reacted for 10, 20, 30, 40, 60, 90, 120, 180, 360 min. After, a small aliquot was taken for ^1^H NMR and GPC analysis of the block copolymer.

### Synthesis of PiStOx_10_ *b*-PEtOx_*n*_ Copolymers

Using PiStOx_10_-*b*-PEtOx_99_ as an example, 1.19 mL of the iStOx stock solution was added to a sealed microwave vial with stirrer bar and purged with N_2_ for 15 min. The vial was then placed in an oil bath and the reaction proceeded for 1 h at 110 °C. After, the vial was removed from the oil bath, allowed to cool and placed under a positive pressure of N_2_ while a small aliquot was taken for ^1^H NMR and GPC analysis of the PiStOx block. Next, EtOx (0.80 g, 8.08 mmol, 100 Eqv) and *n*-heptane (5.33 mL) were added to the microwave vial with stirring. A small aliquot was taken for ^1^H NMR analysis to calculate [EtOx]:[PiStOx]. The nitrogen line was removed, and the microwave vial was placed back into the oil bath for 7 h set at 110 °C. After the reaction was completed, the microwave vial was removed from the oil bath and a small aliquot was taken for ^1^H NMR and GPC analysis of the block copolymer. A small aliquot was also taken and diluted into *n*-dodecane to form a 0.5 wt % solution for DLS, SAXS and TEM analysis.

### Synthesis of PiStOx_10_ *b*-PPrOx_*n*_ Copolymers

Using PiStOx_10_-*b*-PPrOx_76_ as an example, 1.19 mL of the iStOx stock solution was added to a sealed microwave vial with stirrer bar and purged with N_2_ for 15 min. The vial was then placed in an oil bath and the reaction proceeded for 1 h at 110 °C. After, the vial was removed from the oil bath, allowed to cool and placed under a positive pressure of N_2_ while a small aliquot was taken for ^1^H NMR and GPC analysis of the PiStOx block. Next, PrOx (0.73g, 6.46 mmol, 80 Eqv) and *n*-heptane (4.92 mL) were added to the microwave vial with stirring. A small aliquot was taken for ^1^H NMR analysis to calculate [PrOx]:[PiStOx]. The nitrogen line was removed, and the microwave vial was placed back into the oil bath for 3 h set at 110 °C. After the reaction was completed, the microwave vial was removed from the oil bath and a small aliquot was taken for ^1^H NMR and GPC analysis of the block copolymer. A small aliquot was also taken and diluted into *n*-dodecane to form a 0.5 wt % solution for DLS, SAXS and TEM analysis.

### Instrumentation

#### ^1^H Nuclear Magnetic Resonance (NMR)

All spectra were recorded on a Bruker Advance III HD 300 MHz. CDCl3 was used as the solvent and the signal of the residual CHCl3 served as reference for the chemical shift, δ. Data analysis was performed using TopSpin 3.2 software.

#### Gel Permeation Chromatography (GPC)

The measurements were performed using THF (2% TEA and 0.01% BHT) as the eluent. The Agilent Technologies 1260 Infinity instrument was equipped with a refractive index (RI) and 308 nm UV detectors, a PLgel 5 μm guard column, and a PLgel 5 μm mixed D column (300 × 7.5 mm). Samples were run at 1 mL min^–1^ at 40 °C. Poly­(methyl methacrylate) standards (Agilent PMMA calibration kits, M-M-10 and M-L-10 MW range 500–120,000 g/mol) were used for the calibration. Before injection (100 μL), the samples were filtered through a PTFE membrane with 0.2 μm pore size. The data was determined by conventional calibration using Agilent GPC/SEC software and plotted in OriginPro 2022b.

#### Dynamic Light Scattering (DLS)

Measurements were carried out on an Anton Paar Litesizer using a Suprasil quartz cuvette (Hellman, 100-QS, light path of 10.00 mm). Samples were measured at 25 °C at a backscattering measuring angle of 175°. Each sample was measured in triplicate with 30 runs per measurement and 5 min equilibration time between each measurement.

#### Transmission Electron Microscopy (TEM)

Nanostructure solutions were imaged without a staining treatment. The samples were drop-casted on 300 mesh carbon-coated copper TEM grids (Agar Scientific, Stansted, U.K.). After 1 min incubation, excess solution was removed by blotting with filter paper and dried under vacuum before imaging. Bright-field TEM imaging was performed on a JEOL 2100 Plus Transmission Electron Microscope operated at an acceleration voltage of 200 keV. All the images were recorded on a Gatan Orius 11 megapixel digital camera and at least six areas were analyzed.

#### Small Angle X-ray Scattering (SAXS)

Small-angle X-ray scattering (SAXS) measurements were made using a Xenocs Xeuss 2.0 equipped with a microfocus Cu Kα source collimated with Scatterless slits. The scattering was measured using a Pilatus 300k detector with a pixel size of 0.172 mm × 0.172 mm. The distance between the detector and the sample was calibrated using silver behenate (AgC_22_H_43_O_2_), giving a value of 1.196 (3) m. The magnitude of the scattering vector (*q*) is given by *q* = (4π sinθ)/λ, where 2θ is the angle between the incident and scattered X-rays and λ is the wavelength of the incident X-rays. This gave a q range for the detector of 0.003 and 0.3 Å^–1^.

An azimuthal integration of the 2D scattering profile was performed using Xenocs XSACT software and the resulting data corrected for the absorption, sample thickness and background. SAXS patterns were collected at 25 °C for with four repeat one hour collections. These four measurements were then combined to produce a single file with a total counting time of four hours.

## Results and Discussion

CROP of 2-oxazolines in nonpolar solvents have seldom been reported. This is not surprising given that increasing the polarity of the solvent pushes the equilibrium between the oxazolinium and covalent propagating species toward the cationic species, thus leading to an increase in the rate of propagation. Therefore, why would one choose a nonpolar solvent for the CROP of 2-oxazolines? Historically, only one example exists in which 2-methyl-2-oxazoline was polymerized in carbon tetrachloride using benzyl bromide as an initiator. *n*-Heptane was selected as the nonpolar solvent for this CROPISA study. Furthermore, we hypothesized that the high volatility of heptane compared to dodecane would complement the quick drying technique used for TEM sample preparation. However, we later diluted our dispersions in *n*-dodecane for analysis as we found that *n*-heptane evaporated too quickly leading to large polymer aggregates on the TEM grid.

To begin with, a kinetic investigation into the CROP of 2-isosterayl-2-oxazoline (iStOx) was conducted at 110 °C in *n*-heptane at 30 wt % solids (0.72M) using MeOTs as the initiator ([M]:[I] = 60). Figure 1 shows the kinetic data obtained for CROP of iStOx using the above conditions. The sharp increase in molecular weight at low conversion values in Figure 1B and the nonlinear trend of the semilogarithmic plot in Figure 1C are indicative signs of slow initiation. Despite this, a clear molecular weight evolution at each time point can be seen in Figure 1A and relatively narrow molecular weights are achieved at low conversion values highlighting the living nature of CROP in nonpolar media. Given that a short PiStOx block length (DP = 10) will be targeted for the BCPs, we were satisfied in proceeding with the reaction conditions used in the kinetic investigation. Also, conducting CROP at lower temperatures is known to reduce chain transfer events^67^ which is an added benefit of conducting CROP at 110 °C compared to the widely reported 140 °C.

**Figure 1:**
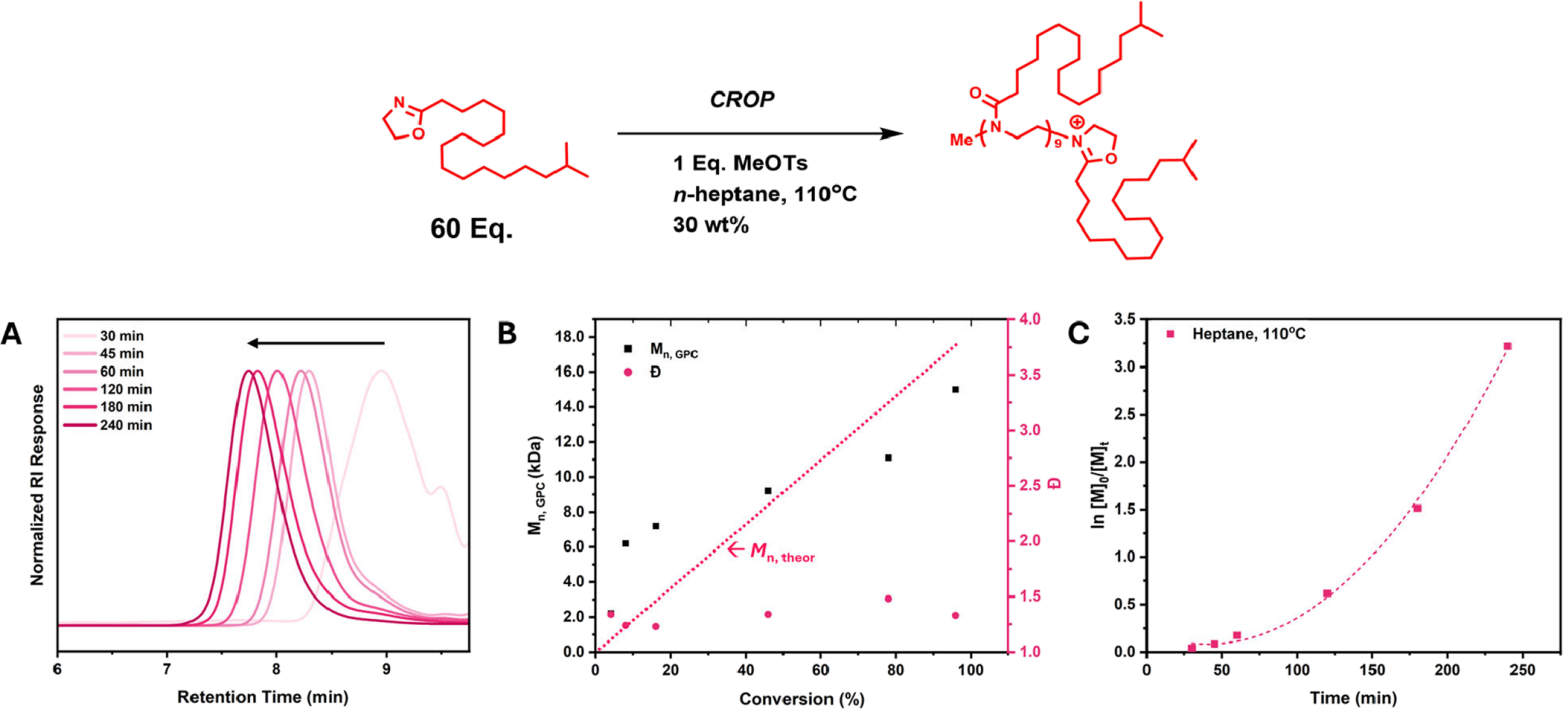
(A) SEC chromatograms of each time interval of the CROP of iStOx in *n*-heptane at 110 °C. (B) Figure illustrating the dependency of molecular weight (*M*_n, SEC_) and dispersity (Đ) on conversion for the CROP of iStOx in *n*-heptane at 110 °C. The disparity between theoretical *M*_n_ and obtained *M*_n_ can be attributed to slow initiation. (C) Semilogarithmic kinetic plot of the CROP of iStOx in *n*-heptane at 110 °C. A breakdown in the linear trend and the presence of a lag period provides additional evidence of slow initiation.

For the initial CROPISA reactions, a short PiStOx_10_ block was first synthesized to full conversion via CROP and then immediately chain extended via sequential monomer addition with 2-ethyl-2-oxazoline (EtOx) in the same conditions used for CROP of iStOx (*n*-heptane, 110 °C) but using a lower reaction concentration of 20 wt % solids. EtOx was selected due to its commercial availability but more importantly, the monomer is soluble in *n*-heptane while the growing polymer block was proven to be insoluble in the same solvent. Figure 2 shows the kinetic data for the CROPISA of PiStOx-*b*-PEtOx ([EtOx]:[PiStOx_10_] = 100) and shows the characteristic enhancement of the rate of propagation around 1 h (Figure 2C). Around the same time, a color change was noted as the colorless solution became translucent with a blue tinge. This rate enhancement and visual change is indicative of PISA and corresponds to the point at which the PEtOx block length reaches a critical length and induces micellization of the block copolymer. EtOx in the *n*-heptane continuous phase is then taken up into the cores of the PiStOx-*b*-PEtOx micelles where the increase in local concentration of the monomer leads to the rate enhancement. Though, the two slopes in the semilogarithmic plot appear to be discontinuous most likely due to the slow initiation of EtOx (Figure 2C). This phenomenon can be further seen in Figure 2B from the sharp increase in *M*_n_ at low conversion with the *M*_n_ plateauing with increased conversion. Over 97% conversion of the EtOx monomer is attained after 6 h with a clear molecular weight evolution seen with increasing conversion (Figure 2A). The dispersity values attained were relatively high for CROP (Đ = 1.33–1.53) indicating a loss of control over the polymerization (Figure 2B).

**Figure 2:**
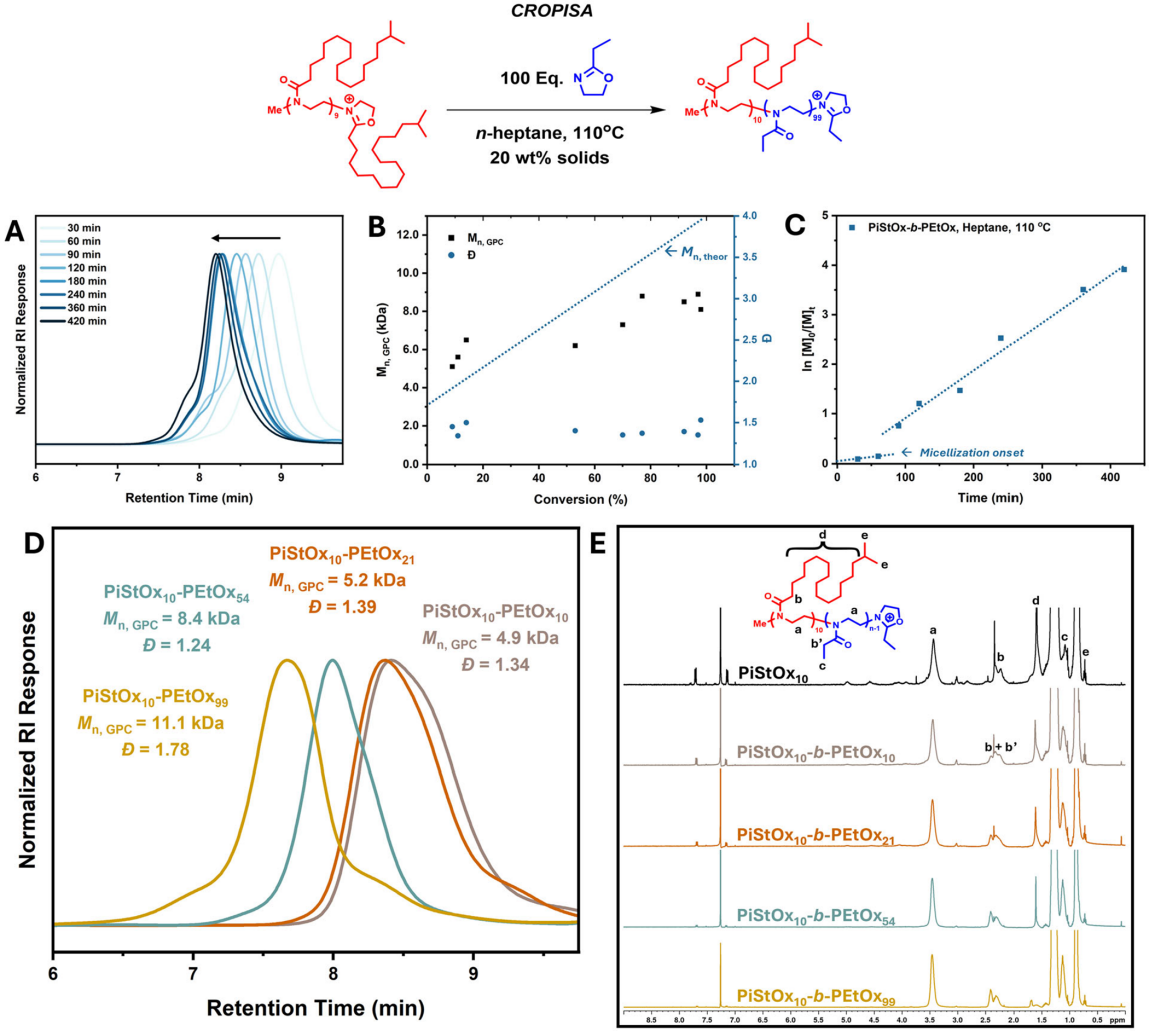
(A) SEC chromatograms of each time interval of the CROPISA of PiStOx-*b*-PEtOx in *n*-heptane at 110 °C. (B) Figure illustrating the dependency of molecular weight (*M*_n, SEC_) and dispersity (Đ) on conversion for the CROPISA of PiStOx-*b*-PEtOx in *n*-heptane at 110 °C. (C) Semilogarithmic kinetic plot of the CROPISA of PiStOx-*b*-PEtOx in *n*-heptane at 110 °C. (D) SEC Chromatograms of the CROPISA of PiStOx_10_-*b*-PEtOx BCPs showing molecular weight and molecular weight distributions for each BCP. Measurements performed using THF (2% TEA and 0.01% BHT) as the eluent. PMMA standards were used for calibration. (E) ^1^H NMR spectra of the PiStOx-*b*-PEtOx block copolymer series (CDCl_3_, 300 MHz).

After the encouraging observation of PISA-like kinetics, a small series of PiStOx_10_-*b*-PEtOx BCPs were synthesized via CROP in *n*-heptane. Varying lengths of PEtOx (DP = 10, 21, 54, 99) were targeted to obtain BCPs with varying solvophilic mass fractions (*f*_PiStOx_ = 77–26%) to see if typical morphologies obtained via PISA can be visualized (e.g., spherical micelles, worms, vesicles). The SEC chromatograms of the PiStOx_10_-*b*-PEtOx series are shown in Figure 2D showing broad molecular weight distributions for each BCP, especially PiStOx_10_-*b*-PEtOx_99_ where a significant high molecular weight shoulder and low molecular weight tailing can be seen. For all BCPs, the appearance of low molecular weight tailing can be seen when comparing the SEC chromatograms of the PiStOx block and the chain extended PiStOx_10_-*b*-PEtOx BCP (Figure S2). This loss of control upon chain extending a long alkyl side chain POx block with a shorter side chain 2-Ox monomer is a phenomenon that has been noted in the literature.^66,68,69^ Interestingly, reversing the order of the block synthesized leads to greater control and more defined BCPs. 1H NMR analysis shows that both blocks of all BCPs went to full conversion proven by the complete disappearance of the EtOx monomer peaks at 3.8 and 4.2 ppm (Figure S1).

The 20 wt % nanostructure dispersions were diluted down to 0.5 wt % and were analyzed by DLS and TEM. Figure 3A shows the DLS traces of each PiStOx_10_-*b*-PEtOx_n_ BCP and in all cases, spherical micelles were formed via CROPISA. Even for PiStOx_10_-b-PEtOx_99_, with a solvophobic mass fraction of *f*_PEtOx_ = 74%, spherical micelles of *D*_h_ = 37 nm were obtained. Complementary TEM images of each BCP are shown in Figure 3B–D and corroborate nicely with the DLS results showing spheres for all PiStOx_10_-*b*-PEtOx BCPs except for PiStOx_10_-b-PEtOx_10_, where the DLS results suggest that the BCP exists as free polymer chains in solution rather than self-assembled nanostructures. Despite the lack of higher order structures seen for PiStOx-*b*-PEtOx, the spheres exhibited excellent colloidal stability at ambient temperature for months, showing no signs of aggregation or sedimentation.

**Figure 3:**
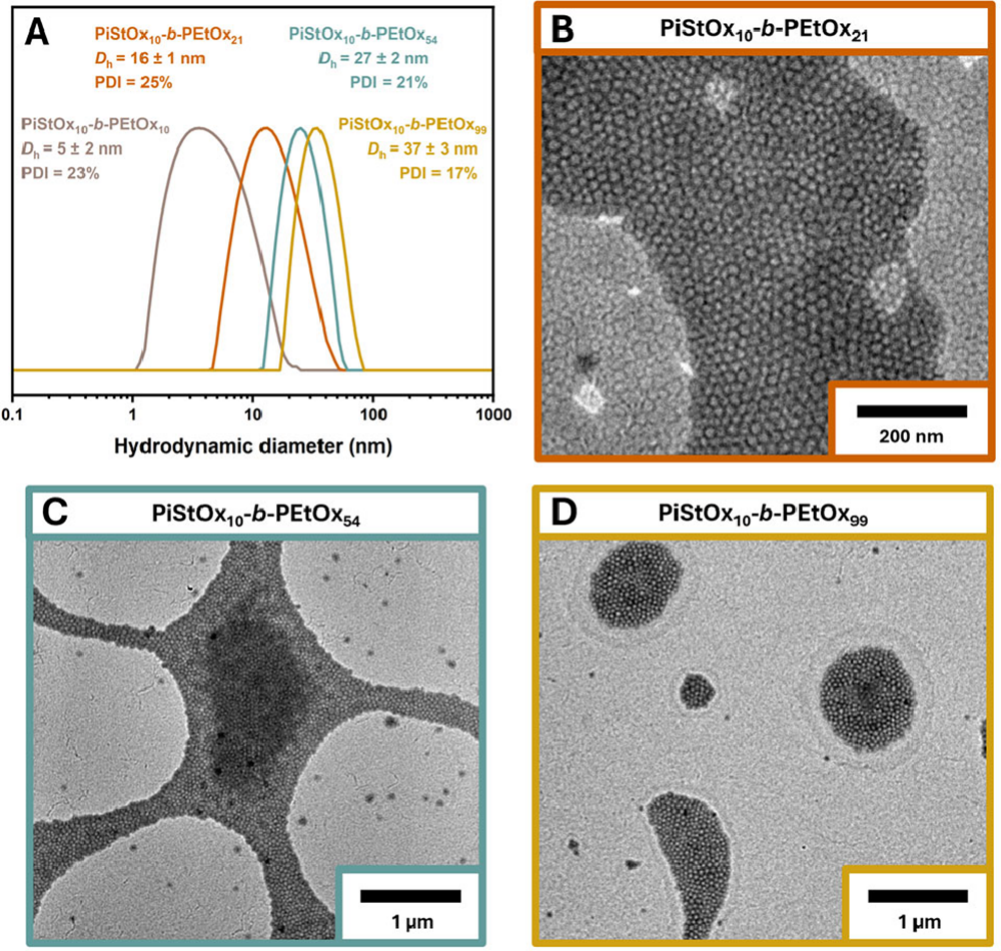
Intensity average DLS traces of the PiStOx-*b*-PEtOx BCP series diluted down to 0.5 wt % in *n*-dodecane (A) Representative TEM images of 0.5 wt % dispersions of PiStOx_10_-*b*-PEtOx_21_ (B), PiStOx_10_-*b*-PEtOx_54_ (C), and PiStOx_10_-*b*-PEtOx_99_ (D).

The formation of kinetically trapped spheres has been reported before for PISA studies in nonpolar media.^16,70,71^ In a recent study by György et al, the group attributed the relatively high *T*_g_ of the core-forming PMMA block to the formation of kinetically trapped spheres in mineral oil.^71^ Lowering the *T*_g_ of the core forming block through copolymerization with 5–10 mol % lauryl methacrylate finally gave access to worms and vesicles. In our case, this should not be an issue due to the polymerization temperature being much higher than the *T*_g_ of PEtOx (110 °C vs ∼60 °C) thus giving the chains sufficient flexibility to facilitate a morphological change. From the same group, Fielding et al. reported that having a relatively large oil-soluble block gives access to kinetically trapped spheres due to sufficient steric stabilization preventing the one-dimensional fusion of spheres to worms.^16^ Lowering the PLMA block from DP = 37 to DP = 17 then allows access to worms and vesicles due to less steric stabilization from the oil-soluble block. In our formulation, we purposely targeted sufficiently low DPs to circumvent this potential problem yet kinetically trapped spheres were the only nanostructure morphology observed. Conducting CROPISA at different solids contents might allow for higher order structures to be obtained. However, across most PISA studies in nonpolar media, there is a weak concentration dependence on the overall block copolymer morphology and hence was not prioritized as a key parameter to change. In lieu of a clear explanation for the formation of kinetically trapped spherical micelles, we postulated that the significant insolubility of PEtOx in *n*-heptane may prevent any sort of chain rearrangement leading to spherical micelles evolving into worms and higher-order morphologies.

Therefore, we opted to tune the solvophobicity of the core-forming block changing PEtOx to PPrOx. We hypothesized that this change to the PISA formulation may provide the system with enough flexibility to access nanostructures beyond that of kinetically trapped spherical micelles. Where the core-forming block is marginally more soluble in *n*-heptane than PEtOx, but still largely insoluble compared to PiStOx to allow self-assembly to occur based on a solubility difference between the two blocks.

A kinetic investigation conducted on the CROPISA of PiStOx-*b*-PPrOx revealed several notable differences compared to the PiStOx-*b*-PEtOx kinetic study. First, the characteristic rate enhancement, as seen earlier in Figure 2C, is absent in the semilogarithmic kinetic plot of PiStOx-*b*-PPrOx (Figure 4C). The entirely linear trend of the kinetic plot suggests chain extension with PrOx proceeds via first-order kinetics indicating significant control over the polymerization. The living nature of the polymerization is further proved by the narrow MWDs obtained (*Đ* = 1.14–1.26), high conversions (>95%) achieved within 3 h, and the linear evolution of molecular weight with increasing conversion that matches to the calculated theoretical *M*_n_ (Figure 4B). The absence of PISA-like kinetics can potentially be ascribed to the similar partition coefficient of PrOx in hexane and the PPrOx core. Due to the increased solubility of PrOx in heptane compared to EtOx, the onset of micellization may not cause the monomer to localize in the core of the micelle unlike the previous CROPISA formulation using EtOx. Despite the absence of PISA-like kinetics, visual changes in the color and consistency of the 20 wt % reaction solutions implied that self-assembly had occurred.

**Figure 4:**
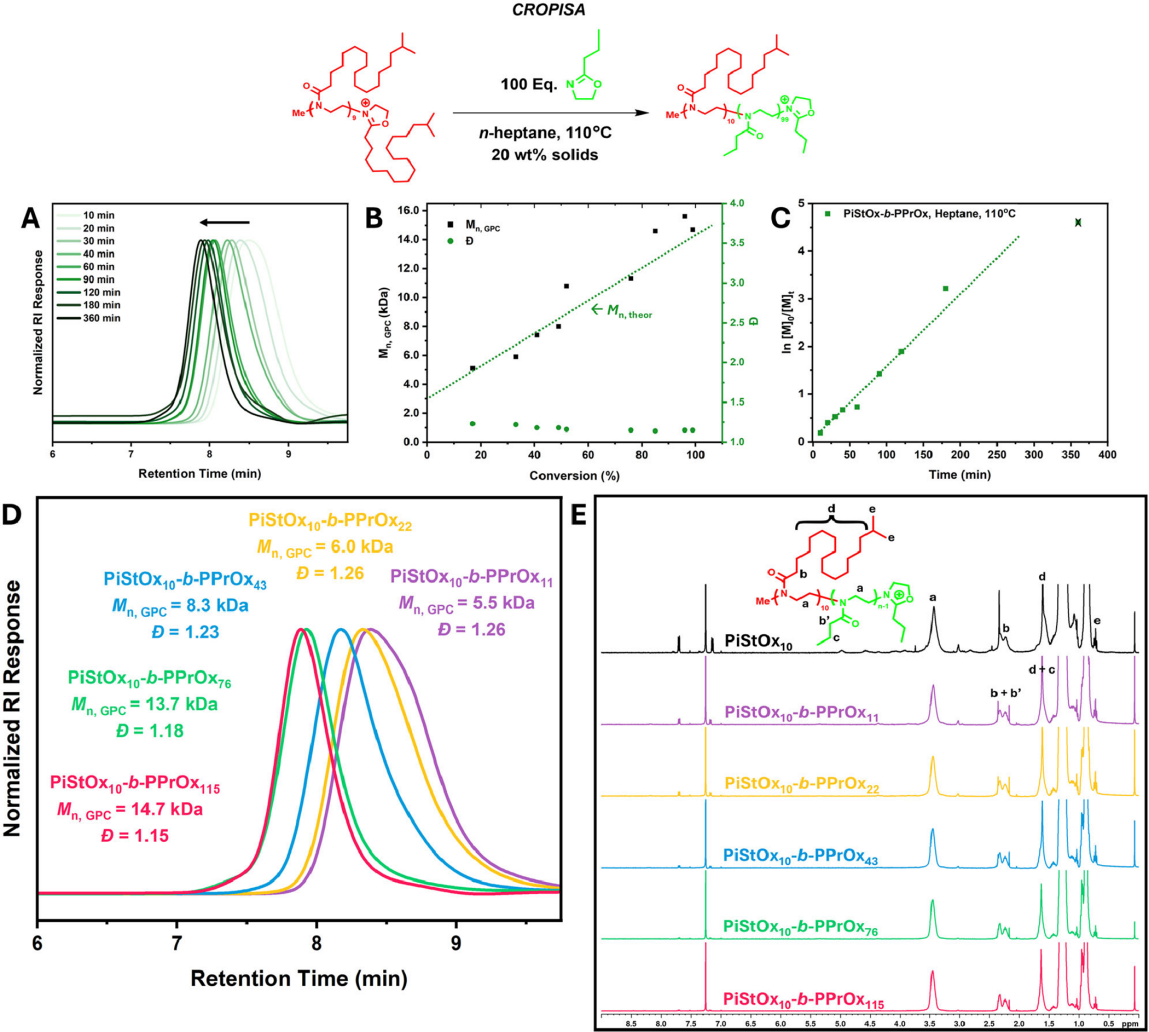
(A) SEC chromatograms of each time interval of the CROPISA of PiStOx-*b*-PPrOx in *n*-heptane at 110 °C (B) Figure illustrating the dependency of molecular weight (*M*_n, SEC_) and molecular weight distribution (Đ) on conversion for the CROPISA of PiStOx-*b*-PPrOx in *n*-heptane at 110 °C. (C) Semilogarithmic kinetic plot of the CROPISA of PiStOx-*b*-PPrOx in *n*-heptane at 110 °C. (D) SEC chromatograms of the CROPISA of PiStOx_10_-*b*-PPrOx_*n*_ BCPs showing molecular weight and molecular weight distributions for each BCP. Measurements performed using THF (2% TEA and 0.01% BHT) as the eluent. PMMA standards were used for calibration. (E) ^1^H NMR spectra of the PiStOx-*b*-PPrOx block copolymer series (CDCl_3_, 300 MHz).

Similarly to PiStOx-*b*-PEtOx, a small series of PiStOx-*b*-PPrOx BCPs were synthesized via CROP at 110 °C in heptane at 20 wt % solids. Varying lengths of PPrOx (DP = 11, 22, 43, 76, 115) were targeted to obtain BCPs with varying solvophilic mass fractions (*f*_iStOx_ = 71–19%) to see if higher-order morphologies beyond spherical micelles can be obtained. Figure 4D shows the SEC chromatograms of the PiStOx-*b*-PPrOx BCP series clearly exhibiting a molecular weight evolution of the series. Narrow molecular weight distributions of the BCP series are observed (*Đ* = 1.15–1.26) and become narrower with increasing lengths of the PPrOx block. Like the PEtOx series, high molecular weight shoulders and low molecular weight tailing can be seen, albeit much less in comparison to the PiStOx-*b*-PEtOx BCPs. Overall, the chain extension of PiStOx with PrOx in *n*-heptane proceeds with remarkably good control. ^1^H NMR analysis shows that both blocks of all BCPs went to full conversion proven by the complete disappearance of the PrOx monomer peaks at 3.8 and 4.2 ppm (Figure S3).

As mentioned earlier, even before DLS, SAXS, and TEM analysis, the visual appearances of the 20 wt % dispersions gave evidence that higher-order morphologies had been obtained. Figure 5A shows an image of the inverted microwave vials of the PiStOx-*b*-PPrOx_*n*_ BCP series after removal from the oil bath. From left to right, PiStOx_10_-*b*-PPrOx_11_ is a clear free-flowing liquid, PiStOx_10_-*b*-PPrOx_22_ and PiStOx_10_-*b*-PPrOx_43_ are translucent free-flowing liquids with the characteristic blue tinge indicative of the presence of nanostructures. Most noticeably, PiStOx_10_-*b*-PPrOx_76_ is a free-standing gel and PiStOx_10_-*b*-PPrOx_115_ is an extremely turbid but free-flowing viscous liquid. After each reaction had finished, aliquots of the 20 wt % dispersions were diluted down to ∼0.5 wt % for DLS, SAXS and TEM analysis. Higher-order morphologies were successfully obtained via CROPISA when replacing the PEtOx block with PPrOx. TEM analysis of each diluted nanoparticle solution shows a variety morphologies obtained as the length of the PPrOx block is increased (Figure 5C–F).

**Figure 5:**
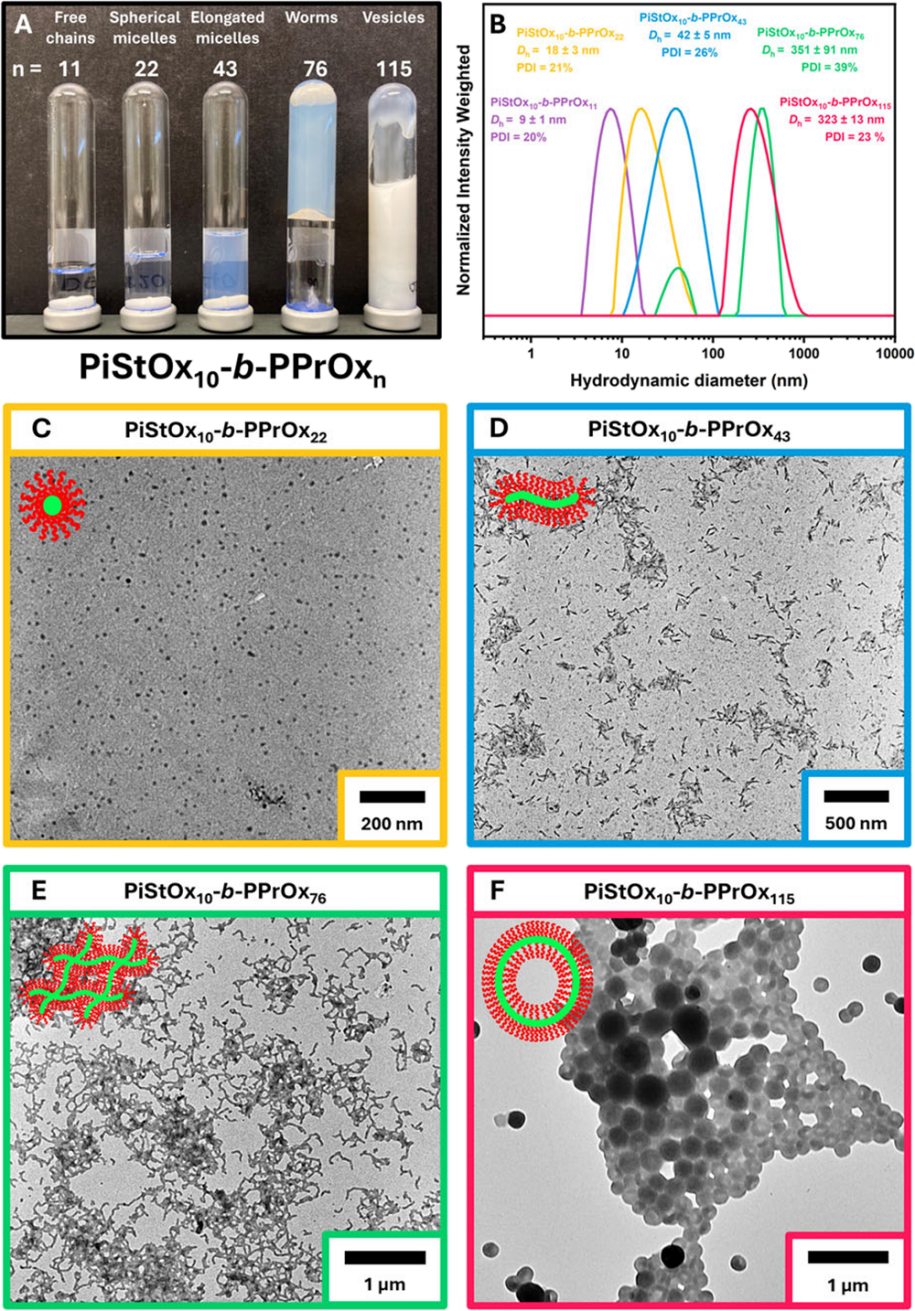
(A) Digital image of the 20 wt % PiStOx_10_-*b*-PPrOx BCP dispersions in *n*-heptane after being removed from the oil bath. (B) Intensity average DLS traces of the PiStOx-*b*-PPrOx BCP series diluted down to 0.5 wt % in *n*-dodecane. Representative TEM images of 0.5 wt % dispersions of PiStOx_10_-*b*-PEtOx_22_ (B), PiStOx_10_-*b*-PEtOx_43_ (C), PiStOx_10_-*b*-PEtOx_76_ (D), and PiStOx_10_-*b*-PEtOx_115_ (E).

Furthermore, DLS analysis shows a clear evolution of the nanoparticle diameters and supports the results seen in the TEM images (Figure 5B). Low DPs of PPrOx (∼10) do not provide the block copolymer chains with sufficient solvophobicity to self-assemble and thus exist as free chains. Increasing the PPrOx block length eventually provides the BCP with sufficient solvophobicity to induce micellization. At PPrOx lengths of DP22 and DP43, spherical and elongated micellar-like structures are obtained, respectively (Figure 5C,D). Increasing the PPrOx chain length further to DP76, the elongated micelles form worm-like structures because of one-dimensional fusion of the PPrOx swollen micelles (Figure 5E). Pushing the PPrOx chain length as high as DP115 eventually causes the worm-like structures to curve and fuse together forming vesicular-like structures with thick bilayers visible in the TEM images (Figure 5F). Representative TEM images of each PiStOx-*b*-PPrOx BCP can be found in the ESI (Figures S5–S8).

Particular interest was paid to the 20 wt % PiStOx_10_-b-PPrOx_76_ dispersion forming the free-standing gel as thermoresponsive worm gels synthesized via PISA in nonpolar media have been well documented in the literature.^71–74^ In an attempt to see whether PiStOx_10_-b-PPrOx_76_ exhibited any thermoresponsive behavior, a sample of the organogel was placed in an oil bath and gradually heated. Due to the highly volatile nature of *n*-heptane, the sample was sealed in a pressure resistant vial to negate solvent evaporation. Figure 6 demonstrates the thermoreversible behavior of the copolymer gel in which heating the 20 wt % PiStOx_10_-b-PPrOx_76_ worm gel in *n*-heptane to 120 °C causes the gel to liquify. Allowing the liquid to cool back to room temperature reforms the copolymer gel. This crude heating and cooling cycle was conducted multiple times with no irreversible gelation/degelation transitions being observed.

**Figure 6:**
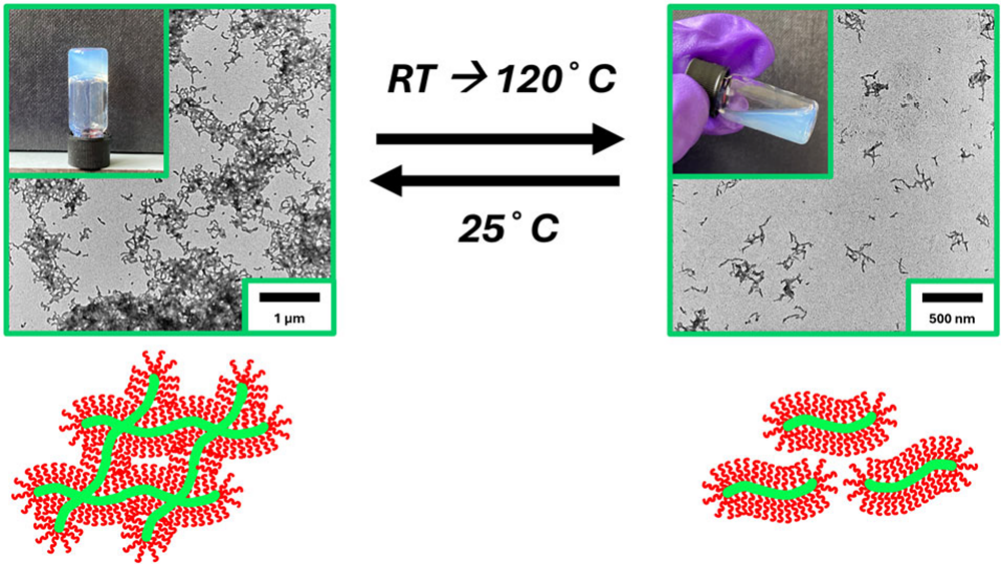
Digital images with the corresponding TEM images of PiStOx_10_-*b*-PEtOx_76_ showing the fully reversible thermoresponsive behavior of the free-standing worm gel. An aliquot the hot, liquified 20 wt % dispersion of PiStOx_10_-*b*-PEtOx_76_ was taken and diluted to 0.1 wt % in hot *n*-dodecane. The diluted dispersion was drop-casted onto a grid to visualize to degelation morphology of the BCP.

An aliquot of the hot 20 wt % copolymer solution was taken and quickly diluted into hot *n*-dodecane to form a 0.1 wt % solution in an attempt to kinetically trap the structures formed from degelation. TEM analysis of this 0.1 wt % solution revealed fragmented structures of the worm-like network, possibly even elongated micelles. This is assuming that the high dilution of the dispersion prevents reformation of the worm-like structures upon cooling. It is plausible that the degelation mechanism occurs via a worm-to-sphere transition, but the high volatility of *n*-heptane compared to other studies utilizing dodecane or mineral oil makes temperature-variable DLS or rheological studies probing this phase transition almost impossible without solvent evaporation.

The 0.5 wt % dispersions of the PiStOx-*b*-PPrOx BCP series were found to be colloidally unstable in *n*-dodecane, with nanostructures gradually aggregating and settling over several weeks at ambient temperature. Initial characterization using DLS and TEM revealed distinct nanostructures depending on the block copolymer composition. SAXS analysis of the BCPs was conducted to corroborate the DLS and TEM findings (Figure 7A–D). PiStOx_10_-*b*-PPrOx_22_ primarily formed spherical micelles, supported by DLS and TEM and expected to exhibit a Guinier region slope ∼ 0 in SAXS analysis. However, SAXS revealed a gradient of −1, suggesting some early aggregation at the time of SAXS measurements (Figure 7A). PiStOx_10_-*b*-PPrOx_43_ and PiStOx_10_-*b*-PPrOx_76_ formed elongated micelles and worm-like micelles, respectively, with SAXS confirming their cylindrical morphologies through slopes of −1 for the Guinier region (Figure 7B,C). PiStOx_10_-*b*-PPrOx_115_ formed vesicles, evident from DLS, SAXS and TEM data, which showed a gradient of −2 and an intervesicle spacing peak at *q* ≈ 0.015 Å^–1^, corresponding to a 45 nm repeat unit (Figure 7D).

**Figure 7:**
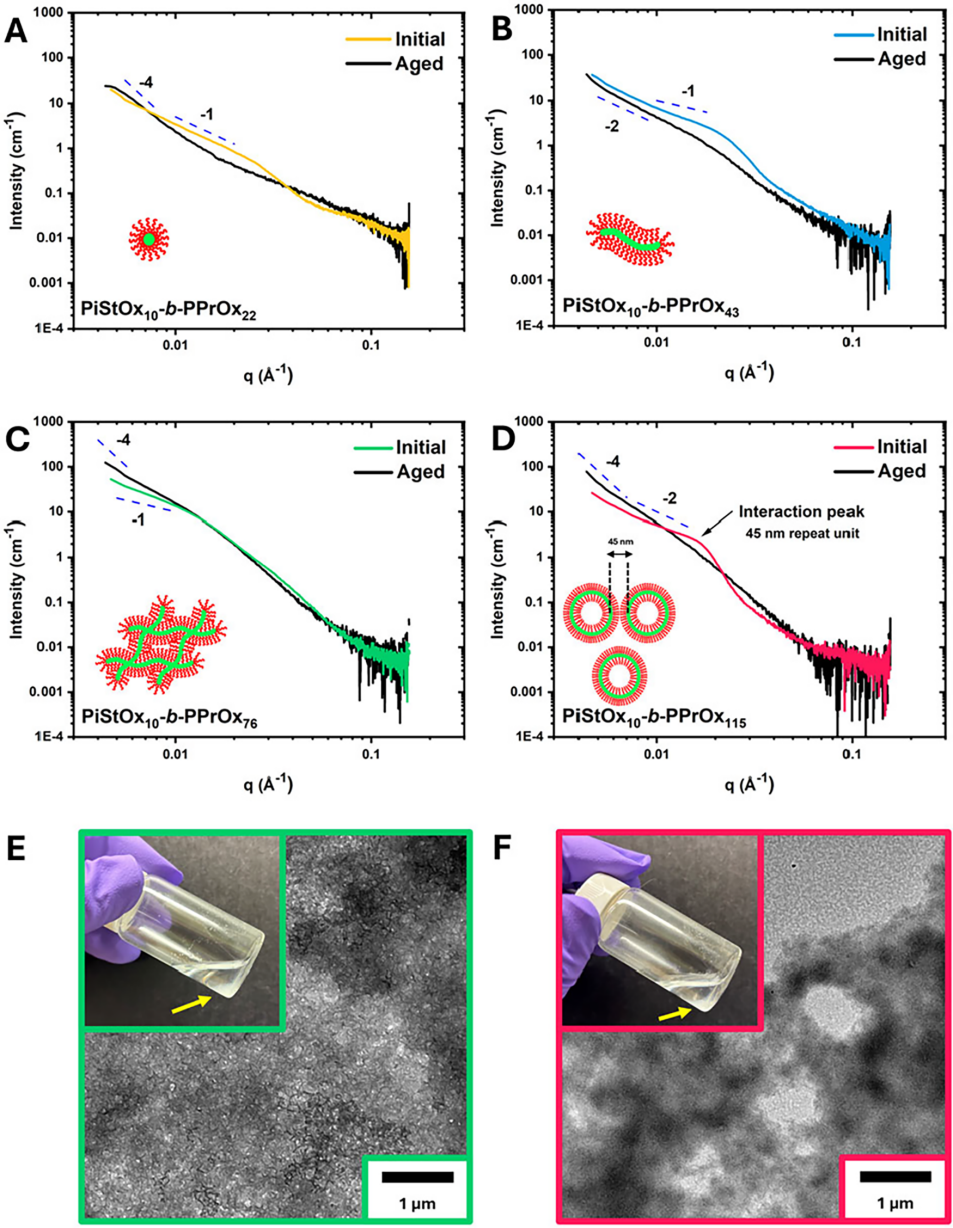
SAXS patterns of initial (colored) and aged (black) samples of PiStOx_10_-*b*-PPrOx_22_ (A), PiStOx_10_-*b*-PPrOx_43_ (B), PiStOx_10_-*b*-PPrOx_76_ (C), and PiStOx_10_-*b*-PPrOx_115_ (D). Representative digital and TEM images of PiStOx_10_-*b*-PPrOx_76_ (E) and PiStOx_10_-*b*-PPrOx_115_ (F) showing the aggregated nature of the aged samples. Yellow arrows are a guide to the eye to show the aggregated polymer that has sedimented on the bottom of the vial.

Over time, these distinct nanostructures transitioned into larger aggregates. SAXS analysis of aged samples revealed a reduction in the features associated with the original morphologies and an increase in low-q scattering, with Guinier region slopes ranging from −2 to −4. TEM images confirmed the loss of distinct structures, showing large, featureless aggregates (Figure 7E,F) while DLS measurements of the aged structures indicated sizes beyond the upper limit of the instrument. Despite attempts to model the SAXS data, high polydispersity in the data complicated precise fitting for core radii and shell thickness. This highlighted the inherent heterogeneity and broad size distributions of the nanostructures. Fitted SAXS curves of the initial and aged structures can be found in the ESI (Figure S9 and Table S1).

These observations suggest that the PiStOx-*b*-PPrOx nanostructures produced via CROPISA are metastable. Initially, they are kinetically trapped in a local energy minimum, stable enough for characterization shortly after preparation. Over time, the kinetically trapped structures rearrange into larger aggregates, representing a thermodynamically favorable state. This transformation occurs as the system overcomes energy barriers that initially stabilized the discrete nanostructures. Ultimately, large aggregates form the most thermodynamically stable state for this system, reflecting a shift from a local to global energy minimum.

## Conclusions

For the first time, successful formation of Poly­(2-oxazoline) BCP higher order nanostructures have been reported including spherical micelles, worms and vesicles via CROPISA. An initial kinetic study of the CROP of iStOx that would form the stabilizing block of the CROPISA formulation was conducted. The kinetic data indicated that slow initiation was occurring although this did not significantly impact the livingness of the polymerization. As a result, short DPs of PiStOx (DP = 10) were targeted for the stabilizing block. Chain extension of the PiStOx_10_ block via sequential monomer addition of EtOx displayed characteristic PISA kinetics with a rate enhancement around 1 h corresponding to the formation of monomer swollen micelles. A small PiStOx_10_-*b*-PEtOx series (DP = 11, 21, 54, 99) was subsequently synthesized via CROP at 20 wt % solids in *n*-heptane yielding block copolymers with a broad range of dispersities (*Đ* = 1.24–1.78). DLS and TEM analysis of diluted 0.5 wt % dispersions showed only spherical micelles (*D*_h_ = 16–37 nm) being produced, even for the highly asymmetric BCP PiStOx_10_-*b*-PEtOx_99_ (*f*_PiStOx_ = 26%). To overcome this unexpected result, the core-forming block was changed from PEtOx to PPrOx. Our rationale was that a core-forming block that is slightly more soluble than PEtOx in *n*-heptane but still solvophobic enough to induce a solubility difference between PiStOx and PPrOx may provide the chains with enough flexibility to allow the one-dimensional fusion of micelles into higher-order structures. A small series of PiStOx_10_-*b*-PPrOx BCPs were synthesized using the exact same conditions as the previous PiStOx_10_-*b*-PEtOx series and yielded a range of nanostructure morphologies (*D*_h_ = 18–323 nm), including spherical micelles, worms, and vesicles which were confirmed by DLS, SAXS, and TEM analysis. Particular attention was paid to the PiStOx_10_-*b*-PPrOx_76_ copolymer organogel that was found to be thermoresponsive on heating to 120 °C and cooling to room temperature which allowed for reversible degelation and gelation over multiple cycles. Despite the challenges in achieving stable nanostructures in nonpolar media, the findings provide a foundation for further work in preparing a variety POx nanostructures using a CROPISA methodology. Future work would include further tuning of the PISA formulations in order to achieve colloidally stable higher order morphologies. Parameters such as different BCP combinations, stabilizing block lengths, and solids contents can be controlled in an attempt to achieve more stable nanostructures. Alternatively, an unsaturated monomer may be utilized for chemical cross-linking of the nanostructures immediately after preparation to physically trap the nanostructures from disassembling.

From an application standpoint, these POx-based nanostructures in nonaqueous media may open new avenues in emerging markets for Poly­(2-oxazoline)­s. For instance, the formation of worm-like and vesicle structures in hydrocarbon solvents holds promise for next-generation oil additives, such as dispersants, friction modifiers, or viscosity index improvers in engine lubricants. Additionally, the thermoresponsive organogel behavior observed in PiStOx_1__0_-b-PPrOx_7__6_ offers potential in smart rheological modifiers or stimuli-responsive soft materials in nonpolar media. The ability to fine-tune morphology via monomer selection also suggests opportunities in controlled release systems where slow diffusion in hydrophobic environments is desirable. As such, CROPISA in nonaqueous media provides a versatile platform for developing advanced materials for industrial applications beyond the traditional aqueous scope.

## Supplementary Material



## Notes

Full experimental details, representative 1H NMR and SEC of each BCP, additional SAXS data, and TEM images of the PiStOx-b-PPrOx series (PDF)

## References

[1] MacFarlane L. R., Shaikh H., Garcia-Hernandez J. D., Vespa M., Fukui T., Manners I. (2021). Functional nanoparticles through π-conjugated
polymer self-assembly. Nature Reviews Materials.

[2] Levin A., Hakala T. A., Schnaider L., Bernardes G. J. L., Gazit E., Knowles T. P. J. (2020). Biomimetic peptide self-assembly
for functional materials. Nature Reviews Chemistry.

[3] Phan H., Taresco V., Penelle J., Couturaud B. (2021). Polymerisation-induced
self-assembly (PISA) as a straightforward formulation strategy for
stimuli-responsive drug delivery systems and biomaterials: recent
advances. Biomaterials Science.

[4] Penfold N. J. W., Yeow J., Boyer C., Armes S. P. (2019). Emerging Trends
in Polymerization-Induced Self-Assembly. ACS
Macro Lett..

[5] Lefley J., Waldron C., Becer C. R. (2020). Macromolecular design
and preparation
of polymersomes. Polym. Chem..

[6] György C., Kirkman P. M., Neal T. J., Chan D. H. H., Williams M., Smith T., Growney D. J., Armes S. P. (2023). Enhanced Adsorption
of Epoxy-Functional Nanoparticles onto Stainless Steel Significantly
Reduces Friction in Tribological Studies. Angew.
Chem., Int. Ed..

[7] Parker B. R., Derry M. J., Ning Y., Armes S. P. (2020). Exploring the Upper
Size Limit for Sterically Stabilized Diblock Copolymer Nanoparticles
Prepared by Polymerization-Induced Self-Assembly in Non-Polar Media. Langmuir.

[8] Rymaruk M. J., Hunter S. J., O’Brien C. T., Brown S. L., Williams C. N., Armes S. P. (2019). RAFT Dispersion
Polymerization in Silicone Oil. Macromolecules.

[9] Derry M. J., Smith T., O’Hora P. S., Armes S. P. (2019). Block Copolymer
Nanoparticles Prepared via Polymerization-Induced Self-Assembly Provide
Excellent Boundary Lubrication Performance for Next-Generation Ultralow-Viscosity
Automotive Engine Oils. ACS Appl. Mater. Interfaces.

[10] Phan H., Cossutta M., Houppe C., Le Cœur C., Prevost S., Cascone I., Courty J., Penelle J., Couturaud B. (2022). Polymerization-Induced Self-Assembly (PISA) for in
situ drug encapsulation or drug conjugation in cancer application. J. Colloid Interface Sci..

[11] Hochreiner E. G., van Ravensteijn B. G. P. (2023). Polymerization-induced
self-assembly
for drug delivery: A critical appraisal. J.
Polym. Sci..

[12] Belluati A., Jimaja S., Chadwick R. J., Glynn C., Chami M., Happel D., Guo C., Kolmar H., Bruns N. (2024). Artificial
cell synthesis using biocatalytic polymerization-induced self-assembly. Nat. Chem..

[13] Blackman L. D., Varlas S., Arno M. C., Houston Z. H., Fletcher N. L., Thurecht K. J., Hasan M., Gibson M. I., O’Reilly R. K. (2018). Confinement
of Therapeutic Enzymes in Selectively Permeable Polymer Vesicles by
Polymerization-Induced Self-Assembly (PISA) Reduces Antibody Binding
and Proteolytic Susceptibility. ACS Central
Science.

[14] Blanazs A., Ryan A. J., Armes S. P. (2012). Predictive Phase
Diagrams for RAFT
Aqueous Dispersion Polymerization: Effect of Block Copolymer Composition,
Molecular Weight, and Copolymer Concentration. Macromolecules.

[15] Blanazs A., Verber R., Mykhaylyk O. O., Ryan A. J., Heath J. Z., Douglas C. W. I., Armes S. P. (2012). Sterilizable
Gels from Thermoresponsive
Block Copolymer Worms. J. Am. Chem. Soc..

[16] Fielding L.
A., Derry M. J., Ladmiral V., Rosselgong J., Rodrigues A. M., Ratcliffe L. P. D., Sugihara S., Armes S. P. (2013). RAFT dispersion
polymerization in non-polar solvents: facile production of block copolymer
spheres, worms and vesicles in n-alkanes. Chemical
Science.

[17] Warren N. J., Mykhaylyk O. O., Mahmood D., Ryan A. J., Armes S. P. (2014). RAFT Aqueous
Dispersion Polymerization Yields Poly­(ethylene glycol)-Based Diblock
Copolymer Nano-Objects with Predictable Single Phase Morphologies. J. Am. Chem. Soc..

[18] Chen M., Li J.-W., Zhang W.-J., Hong C.-Y., Pan C.-Y. (2019). pH- and
Reductant-Responsive Polymeric Vesicles with Robust Membrane-Cross-Linked
Structures: In Situ Cross-Linking in Polymerization-Induced Self-Assembly. Macromolecules.

[19] He W.-D., Sun X.-L., Wan W.-M., Pan C.-Y. (2011). Multiple Morphologies
of PAA-b-PSt Assemblies throughout RAFT Dispersion Polymerization
of Styrene with PAA Macro-CTA. Macromolecules.

[20] Wan W.-M., Hong C.-Y., Pan C.-Y. (2009). One-pot
synthesis of nanomaterials
via RAFT polymerization induced self-assembly and morphology transition. Chem. Commun..

[21] Dai X., Yu L., Zhang Y., Zhang L., Tan J. (2019). Polymerization-Induced
Self-Assembly via RAFT-Mediated Emulsion Polymerization of Methacrylic
Monomers. Macromolecules.

[22] Luo X., Zhao S., Chen Y., Zhang L., Tan J. (2021). Switching
between Thermal Initiation and Photoinitiation Redirects RAFT-Mediated
Polymerization-Induced Self-Assembly. Macromolecules.

[23] Zhang Q., Zeng R., Zhang Y., Chen Y., Zhang L., Tan J. (2020). Two Polymersome Evolution Pathways in One Polymerization-Induced
Self-Assembly (PISA) System. Macromolecules.

[24] Ishizuka F., Kim H. J., Turkovic D., Kuchel R. P., Chatani S., Niino H., Zetterlund P. B. (2023). Synthesis of Hydrophobic Block Copolymer
Nanoparticles in Alcohol/Water Stabilized by Poly­(methyl methacrylate)
via RAFT Dispersion Polymerization-Induced Self-Assembly. Macromolecules.

[25] Kim H. J., Ishizuka F., Chatani S., Niino H., Zetterlund P. B. (2023). Aqueous
RAFT polymerization-induced self-assembly (PISA): amphiphilic macroRAFT
self-assembly vs. monomer droplet nucleation (miniemulsion polymerization). Polym. Chem..

[26] Zhou D., Dong S., Kuchel R. P., Perrier S., Zetterlund P. B. (2017). Polymerization
induced self-assembly: tuning of morphology using ionic strength and
pH. Polym. Chem..

[27] Zhou D., Kuchel R. P., Dong S., Lucien F. P., Perrier S., Zetterlund P. B. (2019). Polymerization-Induced
Self-Assembly under Compressed
CO2: Control of Morphology Using a CO2-Responsive MacroRAFT Agent. Macromol. Rapid Commun..

[28] Kim K. H., Kim J., Jo W. H. (2005). Preparation
of hydrogel nanoparticles by atom transfer
radical polymerization of N-isopropylacrylamide in aqueous media using
PEG macro-initiator. Polymer.

[29] Wan W.-M., Pan C.-Y. (2007). Atom Transfer Radical
Dispersion Polymerization in
an Ethanol/Water Mixture. Macromolecules.

[30] Wang G., Schmitt M., Wang Z., Lee B., Pan X., Fu L., Yan J., Li S., Xie G., Bockstaller M. R., Matyjaszewski K. (2016). Polymerization-Induced
Self-Assembly (PISA) Using ICAR
ATRP at Low Catalyst Concentration. Macromolecules.

[31] Wang J., Wu Z., Wang G., Matyjaszewski K. (2019). In Situ Crosslinking of Nanoparticles
in Polymerization-Induced Self-Assembly via ARGET ATRP of Glycidyl
Methacrylate. Macromol. Rapid Commun..

[32] Kapishon V., Whitney R. A., Champagne P., Cunningham M. F., Neufeld R. J. (2015). Polymerization Induced Self-Assembly of Alginate Based
Amphiphilic Graft Copolymers Synthesized by Single Electron Transfer
Living Radical Polymerization. Biomacromolecules.

[33] Tomasino D. V., Ahmad A., Ahmad T., Salimbeigi G., Dowling J., Lemoine M., Ferrando R. M., Hibbitts A., Branningan R. P., Gibson M. I., Lay L., Heise A. (2024). Surface mannosylation
of dispersion polymerisation derived nanoparticles by copper mediated
click chemistry. Polym. Chem..

[34] Delaittre, G. ; Dire, C. ; Rieger, J. ; Putaux, J.-L. ; Charleux, B. Formation of polymer vesicles by simultaneous chain growth and self-assembly of amphiphilic block copolymers. Chem. Commun. 2009, (20), 2887–2889, 10.1039/b903040a. 19436899

[35] Delaittre G., Save M., Charleux B. (2007). Nitroxide-Mediated
Aqueous Dispersion
Polymerization: From Water-Soluble Macroalkoxyamine to Thermosensitive
Nanogels. Macromol. Rapid Commun..

[36] Delaittre G., Save M., Gaborieau M., Castignolles P., Rieger J., Charleux B. (2012). Synthesis by nitroxide-mediated aqueous
dispersion polymerization, characterization, and physical core-crosslinking
of pH- and thermoresponsive dynamic diblock copolymer micelles. Polym. Chem..

[37] Groison E., Brusseau S., D’Agosto F., Magnet S., Inoubli R., Couvreur L., Charleux B. (2012). Well-Defined
Amphiphilic Block Copolymer
Nanoobjects via Nitroxide-Mediated Emulsion Polymerization. ACS Macro Lett..

[38] Qiao X. G., Dugas P. Y., Charleux B., Lansalot M., Bourgeat-Lami E. (2017). Nitroxide-mediated
polymerization-induced self-assembly of amphiphilic block copolymers
with a pH/temperature dual sensitive stabilizer block. Polym. Chem..

[39] Grubbs R. B. (2011). Nitroxide-Mediated
Radical Polymerization: Limitations and Versatility. Polym. Rev..

[40] Jiang J., Zhang X., Fan Z., Du J. (2019). Ring-Opening
Polymerization
of N-Carboxyanhydride-Induced Self-Assembly for Fabricating Biodegradable
Polymer Vesicles. ACS Macro Lett..

[41] Grazon C., Salas-Ambrosio P., Ibarboure E., Buol A., Garanger E., Grinstaff M. W., Lecommandoux S., Bonduelle C. (2020). Aqueous Ring-Opening
Polymerization-Induced Self-Assembly (ROPISA) of N-Carboxyanhydrides. Angew. Chem., Int. Ed..

[42] Grazon C., Salas-Ambrosio P., Antoine S., Ibarboure E., Sandre O., Clulow A. J., Boyd B. J., Grinstaff M. W., Lecommandoux S., Bonduelle C. (2021). Aqueous ROPISA of α-amino acid
N-carboxyanhydrides: polypeptide block secondary structure controls
nanoparticle shape anisotropy. Polym. Chem..

[43] Boott C. E., Gwyther J., Harniman R. L., Hayward D. W., Manners I. (2017). Scalable and
uniform 1D nanoparticles by synchronous polymerization, crystallization
and self-assembly. Nat. Chem..

[44] Farmer M. A. H., Musa O. M., Armes S. P. (2024). Combining
Crystallization-Driven
Self-Assembly with Reverse Sequence Polymerization-Induced Self-Assembly
Enables the Efficient Synthesis of Hydrolytically Degradable Anisotropic
Block Copolymer Nano-objects Directly in Concentrated Aqueous Media. J. Am. Chem. Soc..

[45] Oliver A. M., Gwyther J., Boott C. E., Davis S., Pearce S., Manners I. (2018). Scalable Fiber-like
Micelles and Block Co-micelles
by Polymerization-Induced Crystallization-Driven Self-Assembly. J. Am. Chem. Soc..

[46] Scanga R. A., Shahrokhinia A., Borges J., Sarault S. H., Ross M. B., Reuther J. F. (2023). Asymmetric
Polymerization-Induced Crystallization-Driven
Self-Assembly of Helical, Rod-Coil Poly­(aryl isocyanide) Block Copolymers. J. Am. Chem. Soc..

[47] Yin R., Sahoo D., Xu F., Huang W., Zhou Y. (2020). Scalable preparation
of crystalline nanorods through sequential polymerization-induced
and crystallization-driven self-assembly of alternating copolymers. Polym. Chem..

[48] Hurst P. J., Rakowski A. M., Patterson J. P. (2020). Ring-opening
polymerization-induced
crystallization-driven self-assembly of poly-L-lactide-block-polyethylene
glycol block copolymers (ROPI-CDSA). Nat. Commun..

[49] Ellis C. E., Garcia-Hernandez J. D., Manners I. (2022). Scalable and Uniform Length-Tunable
Biodegradable Block Copolymer Nanofibers with a Polycarbonate Core
via Living Polymerization-Induced Crystallization-Driven Self-assembly. J. Am. Chem. Soc..

[50] Shen D., Shi B., Zhou P., Li D., Wang G. (2023). Temperature-Dependent
Ring-Opening Polymerization-Induced Self-Assembly Using Crystallizable
Polylactones as Core-Forming Blocks. Macromolecules.

[51] Nuyken O., Pask S. D. (2013). Ring-Opening Polymerization—An Introductory
Review. Polymers.

[52] Wang J., Cao M., Zhou P., Wang G. (2020). Exploration of a Living Anionic Polymerization
Mechanism into Polymerization-Induced Self-Assembly and Site-Specific
Stabilization of the Formed Nano-Objects. Macromolecules.

[53] Verbraeken B., Monnery B. D., Lava K., Hoogenboom R. (2017). The chemistry
of poly­(2-oxazoline)­s. Eur. Polym. J..

[54] Concilio M., Garcia Maset R., Lemonche L. P., Kontrimas V., Song J.-I., Rajendrakumar S. K., Harrison F., Becer C. R., Perrier S. (2023). Mechanism of Action
of Oxazoline-Based Antimicrobial
Polymers Against Staphylococcus aureus: In Vivo Antimicrobial Activity
Evaluation. Adv. Healthcare Mater..

[55] Hayes G., Dias-Barbieri B., Yilmaz G., Shattock R. J., Becer C. R. (2023). Poly­(2-oxazoline)/saRNA
Polyplexes for Targeted and Nonviral Gene Delivery. Biomacromolecules.

[56] Lefley J., Varanaraja Z., Drain B., Huband S., Beament J., Becer C. R. (2023). Amphiphilic
oligo­(2-ethyl-2-oxazoline)­s via straightforward
synthesis and their self-assembly behaviour. Polym. Chem..

[57] Varanaraja Z., Hollingsworth N., Green R., Becer C. R. (2023). Poly­(2-alkyl-2-oxazoline)-Based
Copolymer Library with a Thermoresponsive Behavior in Dodecane. ACS Applied Polymer Materials.

[58] Lefley J., Terracciano R., Varanaraja Z., Beament J., Becer C. R. (2024). Self-Assembly
Behavior of Amphiphilic Poly­(2-ethyl-2-oxazoline)-b-poly­(2-isostearyl-2-oxazoline)
Block Copolymers. Macromolecules.

[59] Terracciano, R. ; Liu, Y. ; Varanaraja, Z. ; Godzina, M. ; Yilmaz, G. ; van Hest, J. C. M. ; Becer, C. R. , Poly­(2-oxazoline)-Based Thermoresponsive Stomatocytes. Biomacromolecules 2024, 25 6050 10.1021/acs.biomac.4c00726. 39146037 PMC11388456

[60] Salgarella A. R., Zahoranová A., Šrámková P., Majerčíková M., Pavlova E., Luxenhofer R., Kronek J., Lacík I., Ricotti L. (2018). Investigation of drug
release modulation from poly­(2-oxazoline) micelles through ultrasound. Sci. Rep..

[61] Sedlacek O., Bardoula V., Vuorimaa-Laukkanen E., Gedda L., Edwards K., Radulescu A., Mun G. A., Guo Y., Zhou J., Zhang H., Nardello-Rataj V., Filippov S., Hoogenboom R. (2022). Influence
of Chain Length of Gradient and Block Copoly­(2-oxazoline)­s on Self-Assembly
and Drug Encapsulation. Small.

[62] Lübtow M. M., Nelke L. C., Seifert J., Kühnemundt J., Sahay G., Dandekar G., Nietzer S. L., Luxenhofer R. (2019). Drug induced
micellization into ultra-high capacity and stable curcumin nanoformulations:
Physico-chemical characterization and evaluation in 2D and 3D in vitro
models. J. Controlled Release.

[63] Le D., Wagner F., Takamiya M., Hsiao I. L., Gil Alvaradejo G., Strähle U., Weiss C., Delaittre G. (2019). Straightforward
access to biocompatible poly­(2-oxazoline)-coated nanomaterials by
polymerization-induced self-assembly. Chem.
Commun..

[64] Finnegan J. R., Davis T. P., Kempe K. (2022). Heat-Induced Living Crystallization-Driven
Self-Assembly: The Effect of Temperature and Polymer Composition on
the Assembly and Disassembly of Poly­(2-oxazoline) Nanorods. Macromolecules.

[65] Finnegan J. R., Pilkington E. H., Alt K., Rahim M. A., Kent S. J., Davis T. P., Kempe K. (2021). Stealth nanorods
via the aqueous
living crystallisation-driven self-assembly of poly­(2-oxazoline)­s. Chemical Science.

[66] Lusiani N., Pavlova E., Hoogenboom R., Sedlacek O. (2025). Cationic Ring-Opening
Polymerization-Induced Self-Assembly (CROPISA) of 2-Oxazolines: From
Block Copolymers to One-Step Gradient Copolymer Nanoparticles. Angew. Chem., Int. Ed..

[67] Monnery B. D., Jerca V. V., Sedlacek O., Verbraeken B., Cavill R., Hoogenboom R. (2018). Defined High Molar Mass Poly­(2-Oxazoline)­s. Angew. Chem., Int. Ed..

[68] Hoogenboom R., Wiesbrock F., Leenen M. A. M., Thijs H. M. L., Huang H., Fustin C.-A., Guillet P., Gohy J.-F., Schubert U. S. (2007). Synthesis
and Aqueous Micellization of Amphiphilic Tetrablock Ter- and Quarterpoly­(2-oxazoline)­s. Macromolecules.

[69] Wiesbrock F., Hoogenboom R., Leenen M., van Nispen S. F. G. M., van der Loop M., Abeln C. H., van den Berg A. M. J., Schubert U. S. (2005). Microwave-Assisted
Synthesis of a 42-Membered Library
of Diblock Copoly­(2-oxazoline)­s and Chain-Extended Homo Poly­(2-oxazoline)­s
and Their Thermal Characterization. Macromolecules.

[70] Darmau B., Rymaruk M. J., Warren N. J., Bening R., Armes S. P. (2020). RAFT dispersion
polymerization of benzyl methacrylate in non-polar media using hydrogenated
polybutadiene as a steric stabilizer block. Polym. Chem..

[71] György C., Neal T. J., Smith T., Growney D. J., Armes S. P. (2022). Tuning
the Glass Transition Temperature of a Core-Forming Block during Polymerization-Induced
Self-Assembly: Statistical Copolymerization of Lauryl Methacrylate
with Methyl Methacrylate Provides Access to Spheres, Worms, and Vesicles. Macromolecules.

[72] Derry M. J., Mykhaylyk O. O., Armes S. P. (2021). Shear-induced alignment of block
copolymer worms in mineral oil. Soft Matter.

[73] Raphael E., Derry M. J., Hippler M., Armes S. P. (2021). Tuning the properties
of hydrogen-bonded block copolymer worm gels prepared via polymerization-induced
self-assembly. Chemical Science.

[74] Fielding L.
A., Lane J. A., Derry M. J., Mykhaylyk O. O., Armes S. P. (2014). Thermo-responsive
Diblock Copolymer Worm Gels in Non-polar
Solvents. J. Am. Chem. Soc..

