# Supplementary material for: Access to Self-Assembled Poly(2-Oxazoline)s through Cationic Ring Opening Polymerization-Induced Self-Assembly (CROPISA)
Source: Macromolecules. 2025 Jul 14;58(15):7744–56. doi: 10.1021/acs.macromol.5c01599 (PMC12356064; doi:10.1021/acs.macromol.5c01599)
Supplement: Supplementary file 1 [file ma5c01599_si_001.pdf]

Electronic Supporting Information:

Access to Self-assembled Poly(2-Oxazoline)s through Cationic Ring Opening Polymerization-induced Self-Assembly (CROPISA)

James Lefley[a], Steven Huband[b], and C. Remzi Becer[a]*

[a] Department of Chemistry, University of Warwick, Coventry, CV4 7AL, United Kingdom

[b] X-ray Diffraction RTP, Department of Physics, University of Warwick, Coventry, CV4 7AL, UK

* Corresponding author: Remzi.Becer@warwick.ac.uk

Table of Contents

Representative NMR of PiStOx$_{10}$-*b*-PEtOx$_{21}$	**2**
GPC Chromatograms of the PiStOx-*b*-PEtOx series	**2**
Representative NMR of PiStOx$_{10}$-*b*-PPrOx$_{22}$	**3**
GPC Chromatograms of the PiStOx-*b*-PPrOx series	**4**
Representative TEM images of 0.5 wt% dispersions of PiStOx$_{10}$-*b*-PPrOx$_{22}$	**5**
Representative TEM images of 0.5 wt% dispersions of PiStOx$_{10}$-*b*-PPrOx$_{43}$	**6**
Representative TEM images of 0.5 wt% dispersions of PiStOx$_{10}$-*b*-PPrOx$_{76}$	**7**
Representative TEM images of 0.5 wt% dispersions of PiStOx$_{10}$-*b*-PPrOx$_{115}$	**8**
Fitted SAXS patterns of the initial and aged 0.5 wt% dispersions	**9**
Fit parameters of SAXS modelling of PiStOx$_{10}$-*b*-PPrOx series	**10**

Appendix

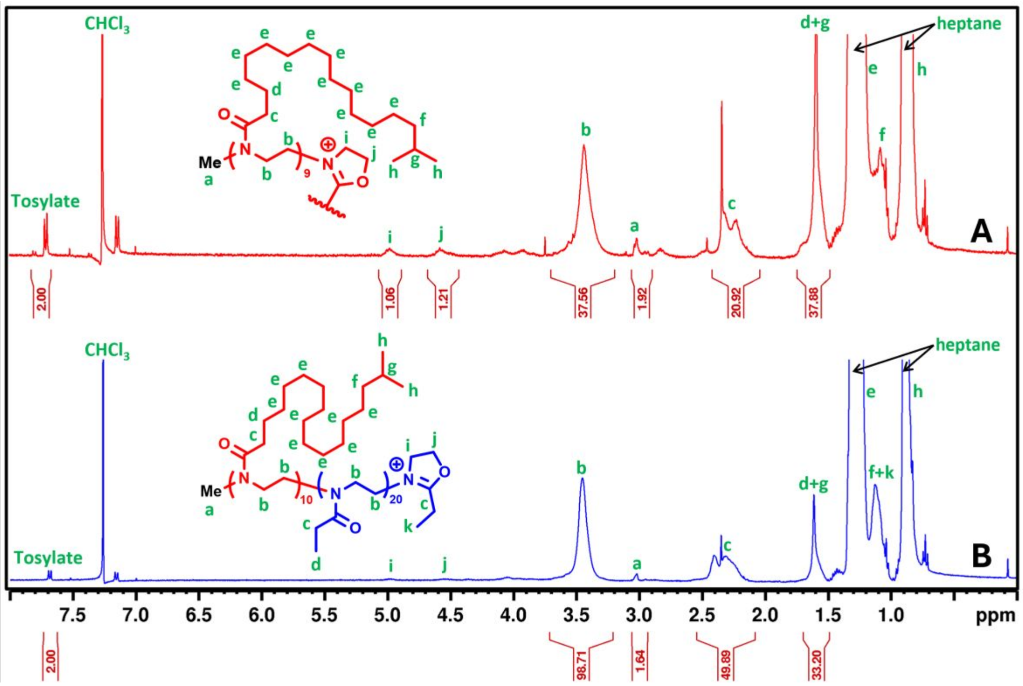

Figure S1. Representative assigned NMR spectra ($CDCl_3$, 300MHz) of PiStOx$_{10}$-*b*-PEtOx$_{21}$ showing full conversion of the PiStOx block (**A**) and full conversion of the PEtOx block yielding the diblock copolymer PiStOx$_{10}$-*b*-PEtOx$_{21}$ (**B**).

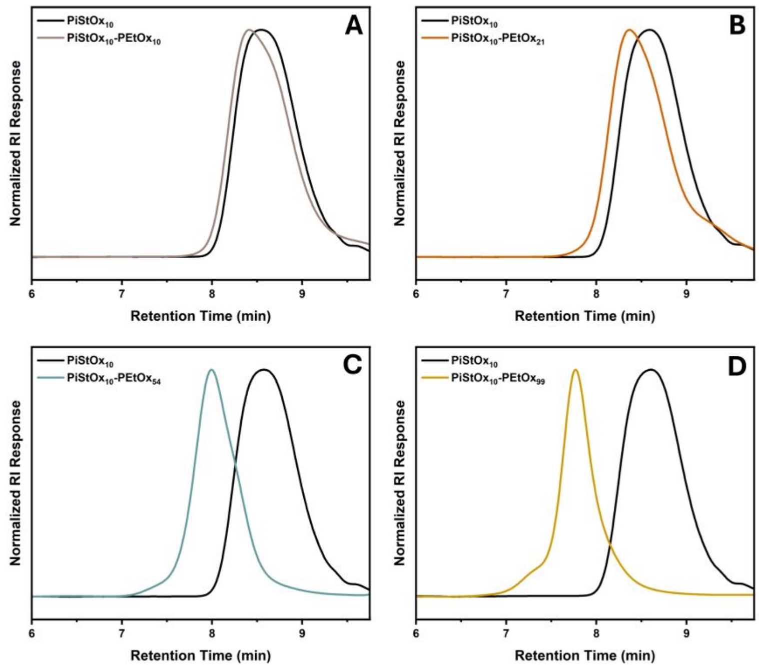

Figure S2. GPC chromatograms of PiStOx$_{10}$-*b*-PEtOx$_{10}$ (**A**), PiStOx$_{10}$-*b*-PEtOx$_{21}$ (**B**), PiStOx$_{10}$-*b*-PEtOx$_{54}$ (**C**), and PiStOx$_{10}$-*b*-PEtOx$_{99}$ (**D**) BCPs showing the first block (black) and the diblock copolymer (colour). Measurements performed using THF (2% TEA and 0.01% BHT) as the eluent. PMMA standards were used for the calibration.

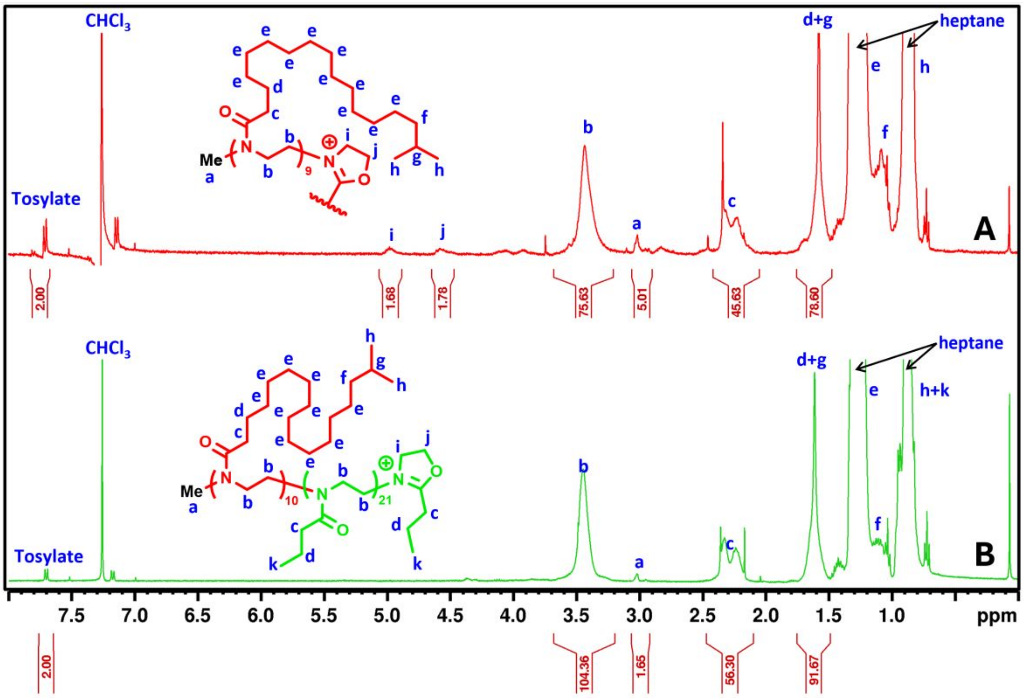

Figure S3. Assigned NMR spectra ($CDCl_3$, 300MHz) of PiStOx$_{10}$-*b*-PPrOx$_{22}$ showing full conversion of the PiStOx block (**A**) and full conversion of the PEtOx block yielding the diblock copolymer PiStOx$_{10}$-*b*-PPrOx$_{22}$ (**B**).

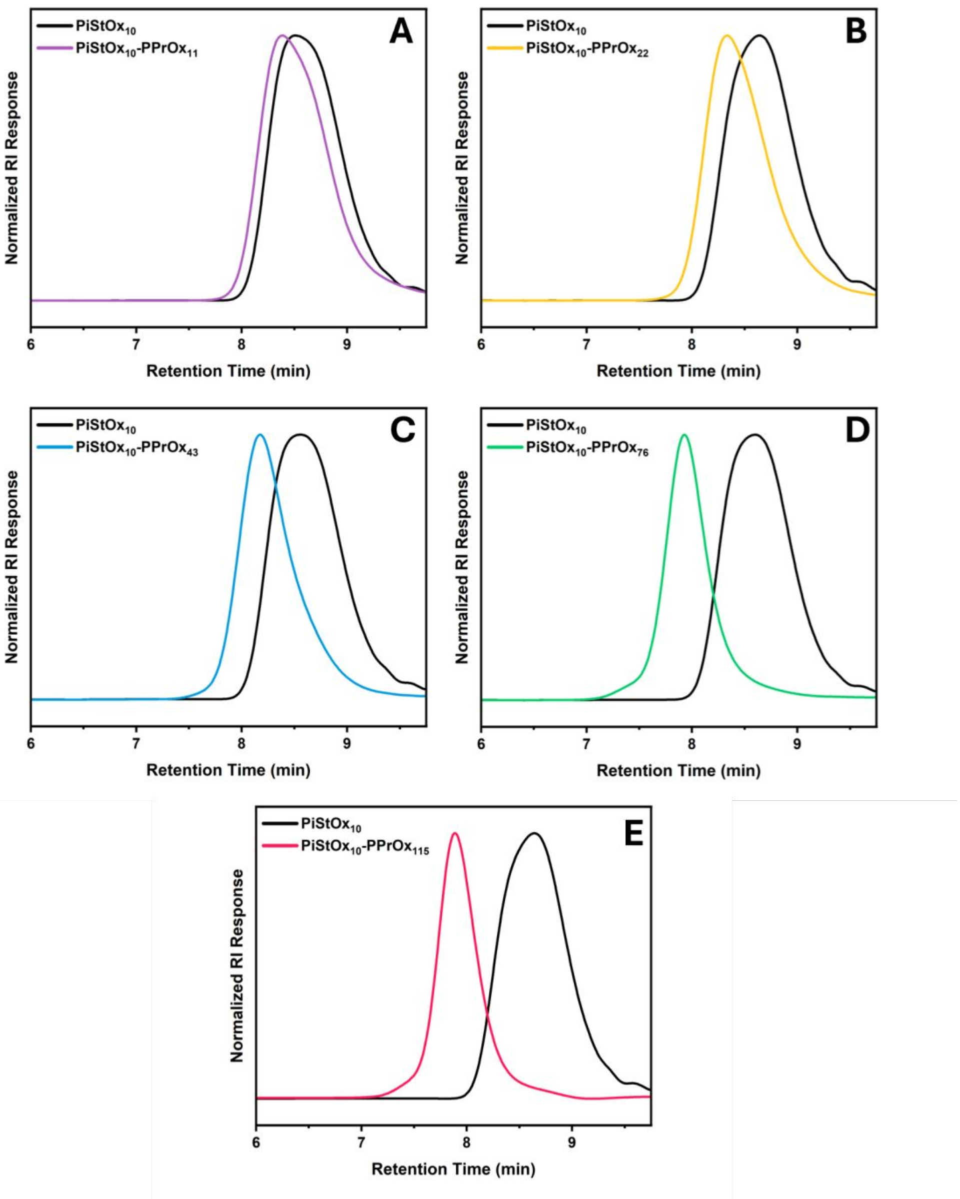

Figure S4. GPC chromatograms of PiStOx$_{10}$-*b*-PPrOx$_{11}$ (**A**), PiStOx$_{10}$-*b*-PPrOx$_{22}$ (**B**), PiStOx$_{10}$-*b*-PPrOx$_{43}$ (**C**), PiStOx$_{10}$-*b*-PPrOx$_{76}$ (**D**), and PiStOx$_{10}$-*b*-PPrOx$_{115}$ BCPs showing the first block (black) and the diblock copolymer (colour). Measurements performed using THF (2% TEA and 0.01% BHT) as the eluent. PMMA standards were used for the calibration.

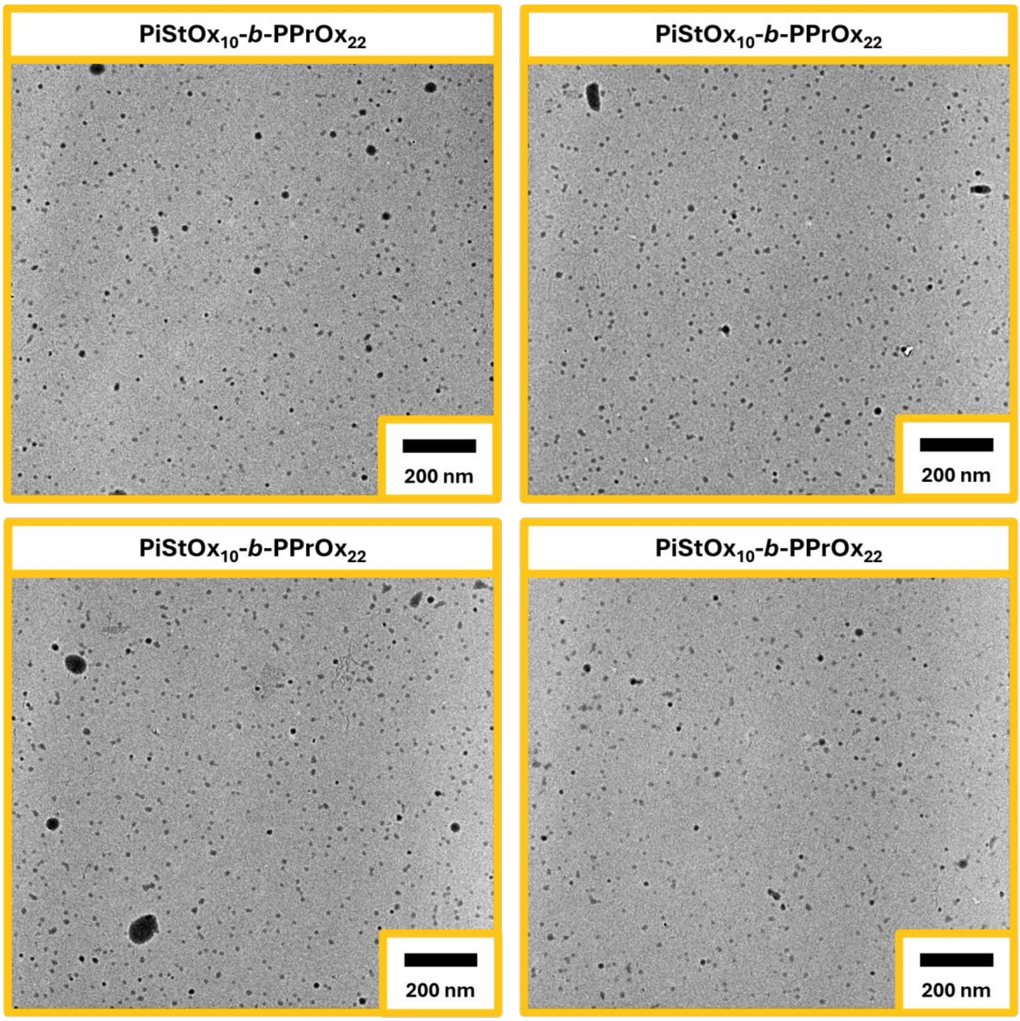

Figure S5. Representative TEM images of 0.5 wt% dispersions of PiStOx$_{10}$-*b*-PPrOx$_{22}$ in *n*-dodecane.

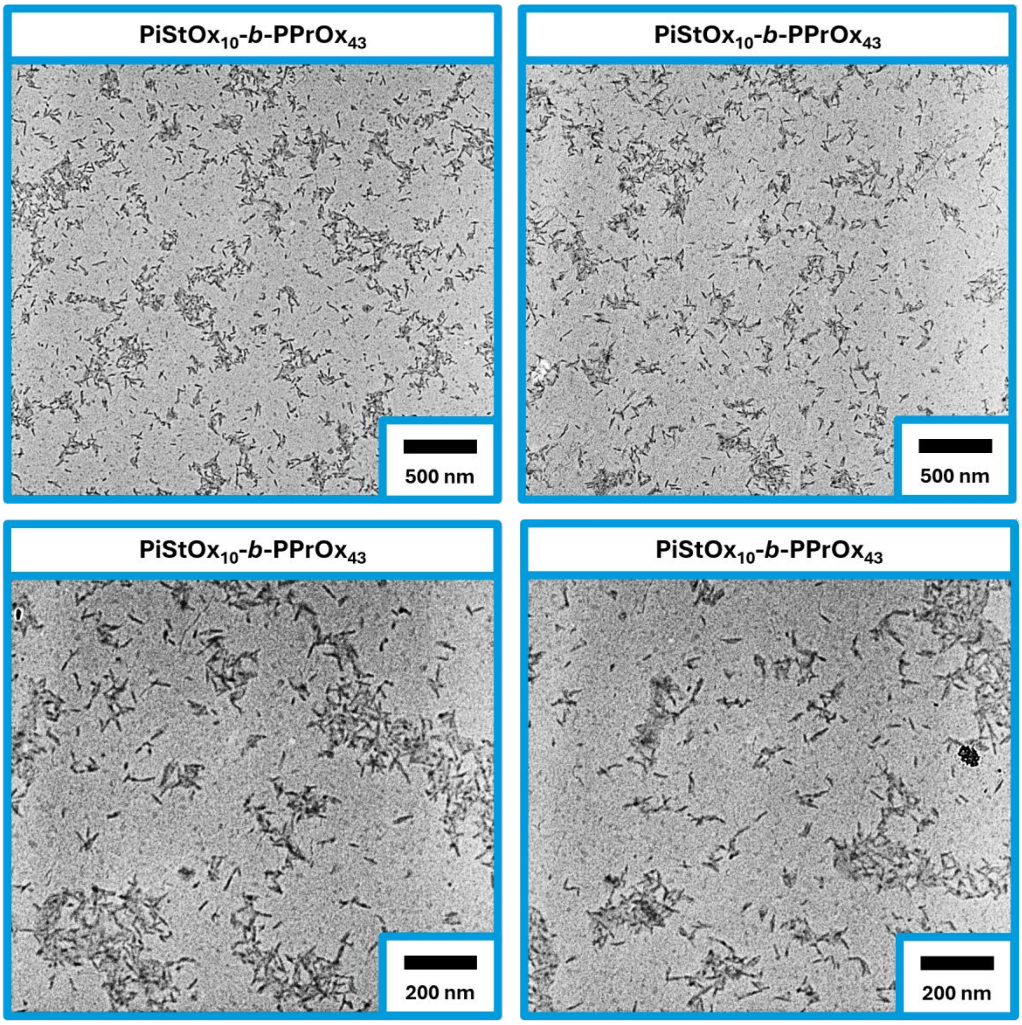

Figure S6. Representative TEM images of 0.5 wt% dispersions of $PiStOx_{10}$-*b*-$PPrOx_{43}$ in *n*-dodecane.

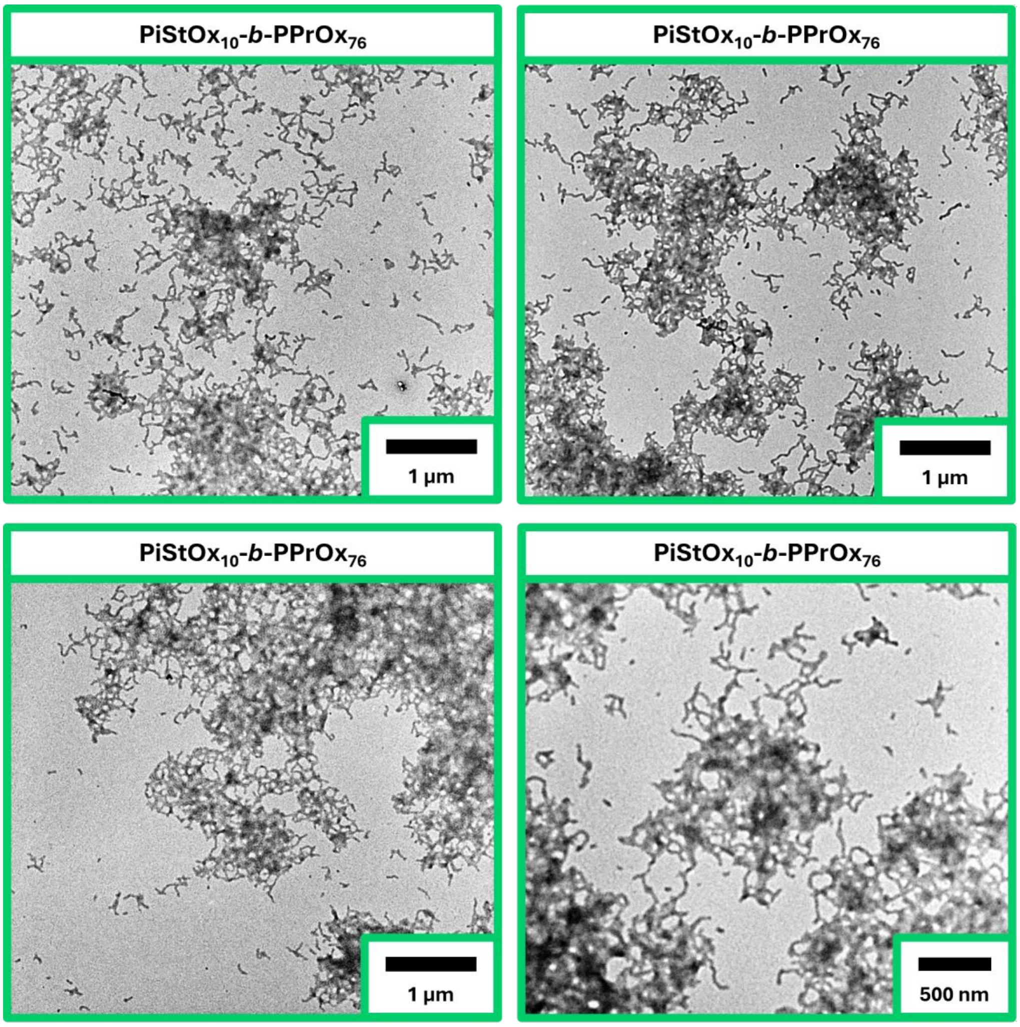

Figure S7. Representative TEM images of 0.5 wt% dispersions of PiStOx$_{10}$-*b*-PPrOx$_{76}$ in *n*-dodecane.

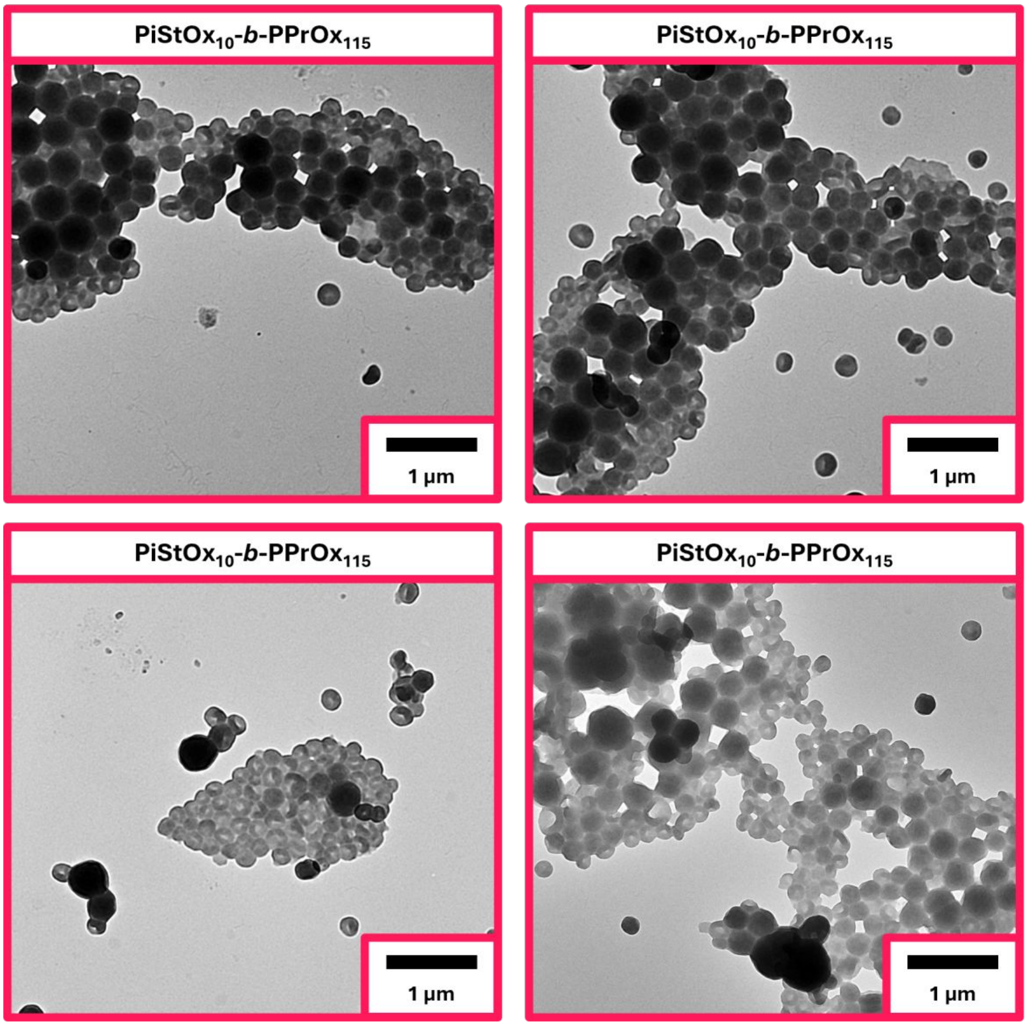

Figure S8. Representative TEM images of 0.5 wt% dispersions of $PiStOx_{10}$-*b*-$PPrOx_{115}$ in *n*-dodecane.

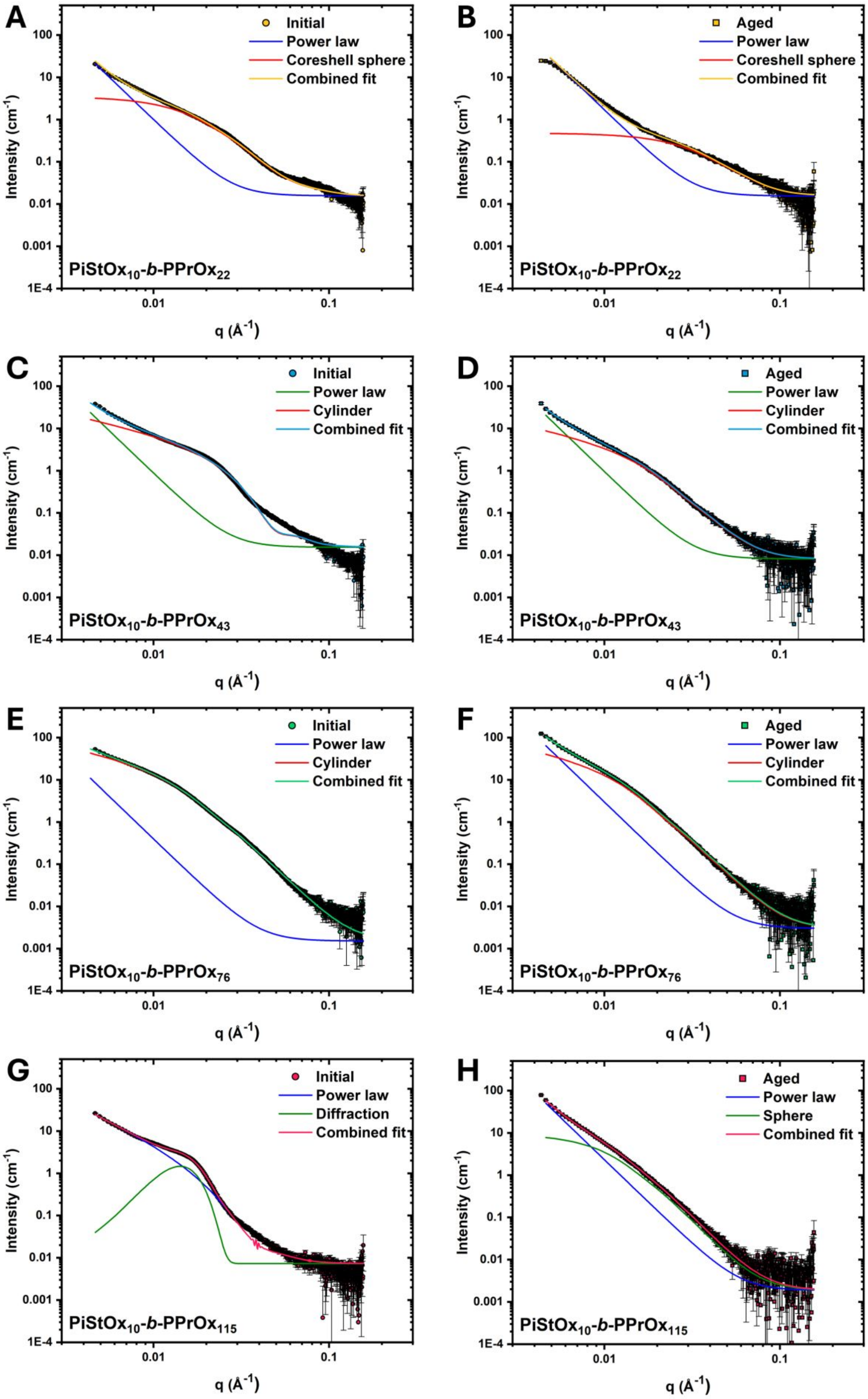

Figure S9. Fitted SAXS patterns of the initial and aged 0.5 wt% dispersions showing the combination of SAXS models in an attempt to model both low and high *q* features of each curve.

Table S1. Fit parameters of SAXS modelling of PiStOx$_{10}$-*b*-PPrOx series. (I) – initial measurement and (A) – aged measurement.

**Polymer**	**Model**	**Core radius (nm)**	**Core radius PDI**	**Shell thickness (nm)**	**Shell thickness PDI**
PiStOx$_{10}$-*b*-PPrOx$_{22}$ (I)	Core-shell sphere + Power law slope	3.0	0.60	3.1	0
PiStOx$_{10}$-*b*-PPrOx$_{22}$ (A)	Core-shell sphere + Power law slope	1.2	0.71	1.8	0
PiStOx$_{10}$-*b*-PPrOx$_{43}$ (I)	Cylinder + Power law slope Length fixed to 300 nm	7.1	0.18	-	-
PiStOx$_{10}$-*b*-PPrOx$_{43}$ (A)	Cylinder + Power law slope Length fixed to 300 nm	1.9	0.89	-	-
PiStOx$_{10}$-*b*-PPrOx$_{76}$ (I)	Cylinder + Power law slope Length fixed to 300 nm	2.5	0.86	-	-
PiStOx$_{10}$-*b*-PPrOx$_{76}$ (A)	Cylinder + Power law slope Length fixed to 300 nm	2.2	1.00	-	-
PiStOx$_{10}$-*b*-PPrOx$_{115}$ (I)	Core-shell (dodecane core) Fixed at 200 nm	-	-	15.0	0.59
PiStOx$_{10}$-*b*-PPrOx$_{115}$ (A)	Sphere + Power law slope	5.4 nm	0.26	-	-
